# Tailored Synthesis of Doped Non‐Layered Oxide Nanosheets Using Designed Solid‐State Surfactants

**DOI:** 10.1002/advs.75720

**Published:** 2026-05-19

**Authors:** Kentaro Ito, Eisuke Yamamoto, Kohei Hayashi, Daiki Kurimoto, Makoto Kobayashi, Minoru Osada

**Affiliations:** ^1^ Department of Materials Chemistry & Institute of Materials and Systems for Sustainability (IMaSS) Nagoya University Nagoya Japan; ^2^ Research Institute for Quantum and Chemical Innovation, Institutes of Innovation for Future Society Nagoya University Nagoya Japan

**Keywords:** doping, nanotechnology, rare earths, surfactants

## Abstract

Precise compositional control of 2D nanosheets is critical for advancing the rational design of 2D functional materials. Solid‐state surfactant templating has recently emerged as a powerful strategy for synthesizing non‐layered 2D oxide nanosheets; however, its broader applicability has been constrained by the lack of rational design principles for multicomponent precursors. Here, we establish a general framework for precursor surfactant design that enables programmable compositional control in non‐layered 2D oxide nanosheets. Using cerium oxide doped with a range of rare‐earth elements (La, Pr, Sm, Gd, Yb, Y, and Sc) as a model system, we show that precursor crystal compositions are determined not only by nominal stoichiometry but also by selective incorporation governed by ionic radii. In contrast, the resulting nanosheet compositions deviate systematically due to differences in dopant reactivity during condensation, which correlates with the hydrolysis tendency of the metal cations. By rationally balancing these competing incorporation and reaction effects, nanosheets with targeted compositions are achieved. This work elevates solid‐state surfactant templating from an empirical method to a designable synthetic platform, offering broadly applicable principles for the synthesis of compositionally programmed 2D oxide nanosheets.

## Graphical abstract

This work reports a solid‐state surfactant templating method that enables precise doping of non‐layered oxide nanosheets. Using ceria and rare‐earth elements as model systems, we established design principles for solid‐state surfactants containing multiple elements. These designed surfactants enabled the synthesis of rare‐earth‐doped ceria nanosheets with controlled dopant concentrations.

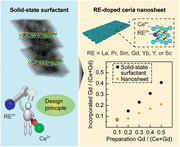

## 1 Introduction

Engineering the properties of oxide materials through structural and compositional tuning is the heart of advances in electronic and energy materials. In particular, doping, the introduction of heteroatoms into the lattice, is a widely employed technique that enables modification of oxide material properties through inducing defects and tuning electronic structures. In recent years, oxide nanomaterials have attracted growing attention, and material design strategies that combine doping with fabricating nanostructures have been explored [1, 2, 3]. It is known that even small deviations in dopant concentration can lead to pronounced and often unpredictable changes in functionality [4, 5]. Therefore, precise doping is essential for engineering the properties of oxide materials with desired nanostructures.

The doping for oxide nanosheets is crucial because of their characteristics originating from their two‐dimensional structure, such as unique carrier transport properties and abundant active sites [6]. Nevertheless, there are difficulties in simultaneously realizing precise compositional control and fabricating two‐dimensional morphologies. Although the exfoliation of layered oxides enables precise doping of nanosheets through the compositional design of parent layered compounds [7, 8, 9], this approach is inherently limited to compositions that form stable layered structures. Recently, various bottom‐up approaches have been developed to produce nanosheets from oxides with non‐layered structures, thereby overcoming compositional limitations [10, 11, 12, 13]. However, examples in which these methods have been used to precisely and systematically tune the properties of oxide nanosheets through compositional control remain very limited [14]. Although composition design has been achieved for selected target compositions, systematic control over nanosheet composition has been demonstrated mainly in liquid‐metal‐based approaches [15]. In such methods, the accessible materials space is limited by the choice of liquid metals or alloys, and alloying can alter the composition of the interfacial oxide [16]. Therefore, despite their promise for realizing attractive properties, a general strategy for the precise and systematic compositional design of non‐layered oxide nanosheets over a wide composition range remains underdeveloped.

As a novel variation of the bottom‐up synthesis method, we recently developed a solid‐state surfactant templating method, which has potential for the precise design of nanosheets [17]. Our approach provides nanosheets via a three‐step process: (1) preparation of precursor solid‐state surfactant crystals, which are composites of surfactants and metal species; (2) transformation of the metal species into reactive clusters within the interlayer spaces by exposing the precursor surfactants to humid ammonia vapor; and (3) assembly of these clusters on the surfactant templates to form nanosheets by aging of the obtained surfactants with clusters in formamide. This method has significant potential for designing nanosheets through stepwise control of each reaction process. In particular, compositional design of the precursor solid‐state surfactants is expected to enable precise control over the composition of the resulting nanosheets. However, there were a few studies on the chemical nature of solid‐state surfactants [18, 19, 20], and the factors governing their compositions have not been systematically investigated, nor has precise doping ever been realized.

In this study, we investigated the significant factors governing the compositional control of solid‐state surfactants, and heteroatom‐doped nanosheets with tailored doping concentrations were synthesized by designing the compositions of precursor solid‐state surfactants. Ce and rare‐earth elements (La, Pr, Sm, Gd, Yb, Y, and Sc) were selected as model systems to clarify which metal species are preferentially incorporated into the surfactants and to elucidate the factors underlying metal incorporation into surfactant crystals. The designed solid‐state surfactant crystals were subsequently subjected to humid ammonia vapor treatment to prepare doped ceria clusters, and their compositional changes were investigated. The obtained intermediate compounds containing ceria clusters were immersed in formamide to obtain rare‐earth‐doped ceria nanosheets, and the variations in composition depending on the type of dopant were clarified. In addition, we demonstrated that rare‐earth doping is effective for controlling the properties of ceria nanosheets. This work establishes a general framework for achieving precise doping in non‐layered nanosheet systems.

## 2 Results and Discussion

### 2.1 Preparation of Solid‐State Surfactant Crystals Containing Ce and Gd

**FIGURE 1:**
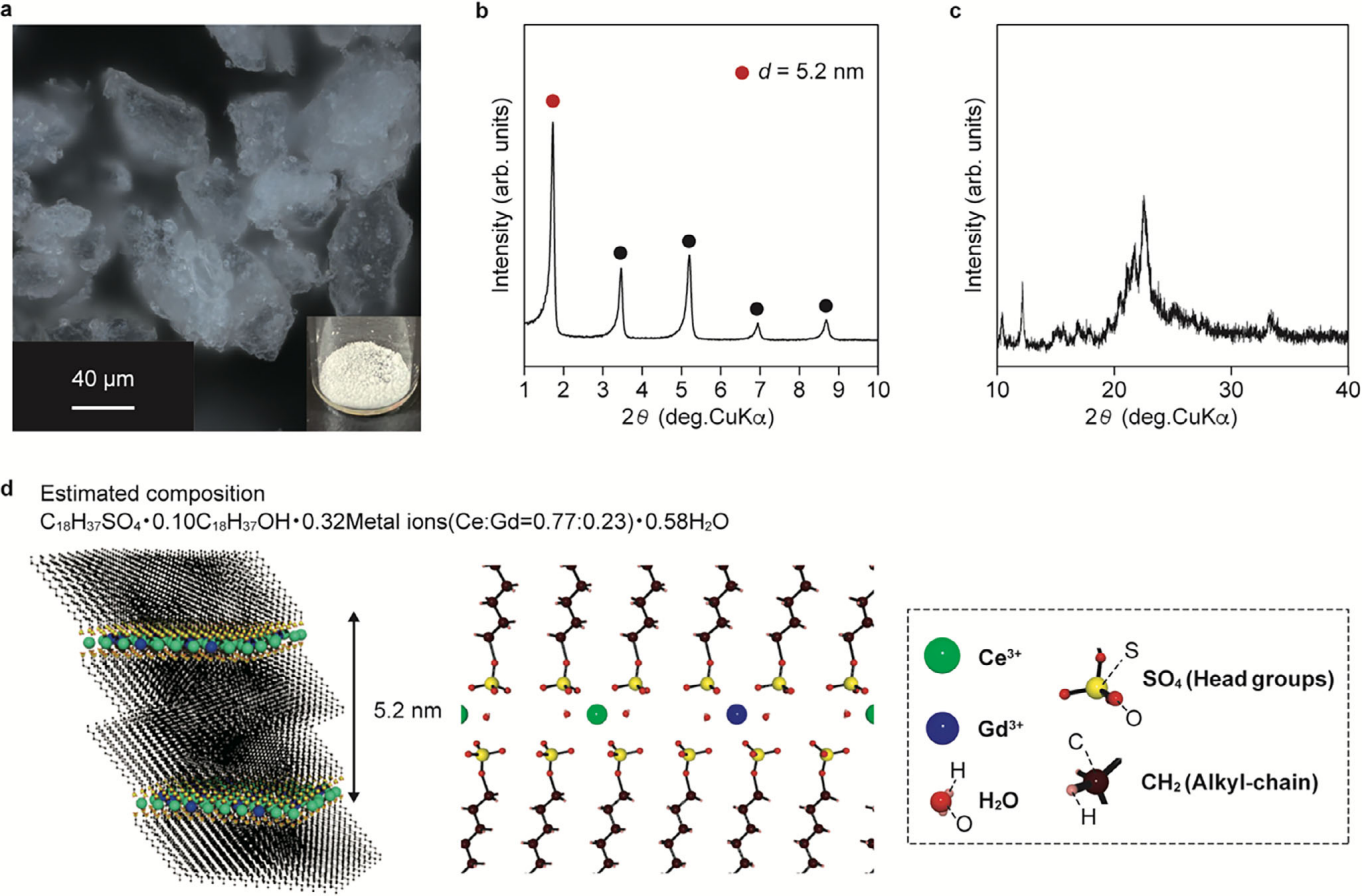
Synthesis of solid‐state surfactant crystals containing both Ce and Gd. (a) Optical microscope image and (inset) appearance of the solid‐state surfactant crystals. (b, c) XRD patterns (b: 1–10°, c: 10–60°) of the solid‐state surfactant crystals. (d) Estimated composition and predicted structure of solid‐state surfactants synthesized with the preparation ratio of Ce:Gd = 0.70:0.30.

Solid‐state surfactants were synthesized by mixing an aqueous solution of metal nitrates with a sodium octadecylsulfate aqueous solution. Mixed solutions of cerium nitrate and other lanthanoid nitrates were used to incorporate both Ce and other metal cations into the surfactants. We employed the Gd ions as a typical example of metals because Gd is known as one of the best dopants to improve the oxygen ionic conductivity of ceria [21]. The preparation molar concentration of metal cations was set 14 times greater than that of sodium octadecylsulfate to prevent the contamination of Na ions into the resulting compounds. The preparation molar ratio of Ce and Gd was adjusted to Ce:Gd = 0.70:0.30. The synthesized compounds were obtained as white crystals, as observed in the optical image (Figure 1a).

To confirm the formation of the solid‐state surfactant, we conducted structural and compositional analyses. First, we investigated the structure and morphology of the solid‐state surfactant crystal by X‐ray diffraction (XRD) measurement and transmission electron microscopy (TEM). The XRD pattern of the crystal showed a series of {00*l*} peaks at 1.7°, 3.4°, 5.1°, 6.9°, and 8.6° (The *d*‐values were 5.2, 2.6, 1.7, 1.3, and 1 nm, respectively.), indicating the formation of lamellar structures (Figure 1b). Additionally, there were peaks around 20°, originating from alkyl chain packing (Figure 1c), strongly suggesting the formation of the *L*_c_ lamellar phase. The TEM image exhibited clear periodic contrasts, confirming the presence of lamellar structures (Figure S1a). The spacing between these periodic contrasts was approximately 5.2 nm, consistent with the XRD results. From these results, the formation of lamellar structures with the *L*_c_ phase was confirmed.

Then, the composition of the solid‐state surfactant crystal was investigated by energy‐dispersive X‐ray fluorescence spectroscopy (EDXRF), inductively coupled plasma atomic emission spectroscopy (ICP‐AES), scanning electron microscopy (SEM)‐energy dispersive X‐ray spectroscopy (EDS), X‐ray photoelectron spectroscopy (XPS), Fourier transform infrared spectroscopy (FT‐IR), thermogravimetric (TG) analysis, and carbon‐hydrogen‐nitrogen‐sulfur (CHNS) analysis. EDXRF analysis showed the peaks which can be assigned to Ce Lα (4.8 keV), Gd Lα (6.1 keV), and S Kα (2.3 keV), and the molar ratio of metals in the surfactant crystal was calculated to Ce:Gd = 0.77:0.23 (Figure S1b). ICP‐AES analysis detected only a negligible amount of Na, indicating that the use of excess metal cations effectively suppressed Na incorporation during the formation of the surfactant crystal. The SEM image, corresponding EDS spectrum, and EDS mappings showed the homogeneous distribution of Ce, Gd, and S (Figure S1c–g). The XPS spectra showed peaks assigned to Ce 3d_3/2_ (895–915 eV), Ce 3d_5/2_ (875–890 eV), Gd 4d_3/2_ (145–150 eV), Gd 4d_5/2_ (135–145 eV), and S 2p_3/2_ (168 eV), also confirming the presence of these elements (Figure S2a–c). Notably, the XPS spectrum in the range of 870–925 eV exhibited peaks (v^0^, v, u^0^, and u) attributed only to trivalent Ce, indicating Ce was incorporated as Ce^3+^ ions [22]. FT‐IR spectroscopy exhibited characteristic bands corresponding to C–H stretching vibrations (2849, 2916, and 2954 cm^−^^1^) and sulfate groups (1100 cm^−^^1^), confirming that the molecular structure of the surfactants was retained after the reactions with metal cations (Figure S2d) [23]. Finally, we calculated the composition of the solid‐state surfactant quantitatively by using the results from TG, CHNS, and EDXRF analyses. TG analysis indicated a 2.4% mass loss below 150 °C, attributed to water evaporation. Notable weight losses were further observed between 150 °C and 800 °C, resulting from the stepwise decomposition that is characteristic of inorganic–organic hybrid compounds (Figure S2e). The residue (12.6 wt.%) was attributed to ceria doped with Gd, confirmed by XRD. CHNS analysis revealed that the surfactant crystal contained carbon (54.43 wt.%), hydrogen (9.57 wt.%), and sulfur (7.34 wt.%) (Table S1). From these results, we estimated the composition of the solid‐state surfactant as C_18_H_37_SO_4_·0.10C_18_H_37_OH·0.32Metal ions (Ce:Gd = 0.77:0.23)·0.58H_2_O (Table S1). The octadecanol, described as C_18_H_37_OH, is the impurity from the reagent. These results indicated the formation of solid‐state surfactant crystals (Figure 1d).

It is important to consider the incorporated ratio of Ce and Gd into the surfactant crystals for rational design. The molar ratio of Ce to Gd in the solid‐state surfactants was 0.77:0.23, whereas the initial preparation ratio was Ce:Gd = 0.70:0.30. The preferential incorporation of Ce indicated that the metal composition of surfactant crystals did not simply follow the preparation ratio. Therefore, it is essential to elucidate the underlying factors which govern the metal incorporation by comparing various synthesis conditions, preparation ratios, and different dopant species.

### 2.2 Metal Composition Control of Solid‐State Surfactants

**FIGURE 2:**
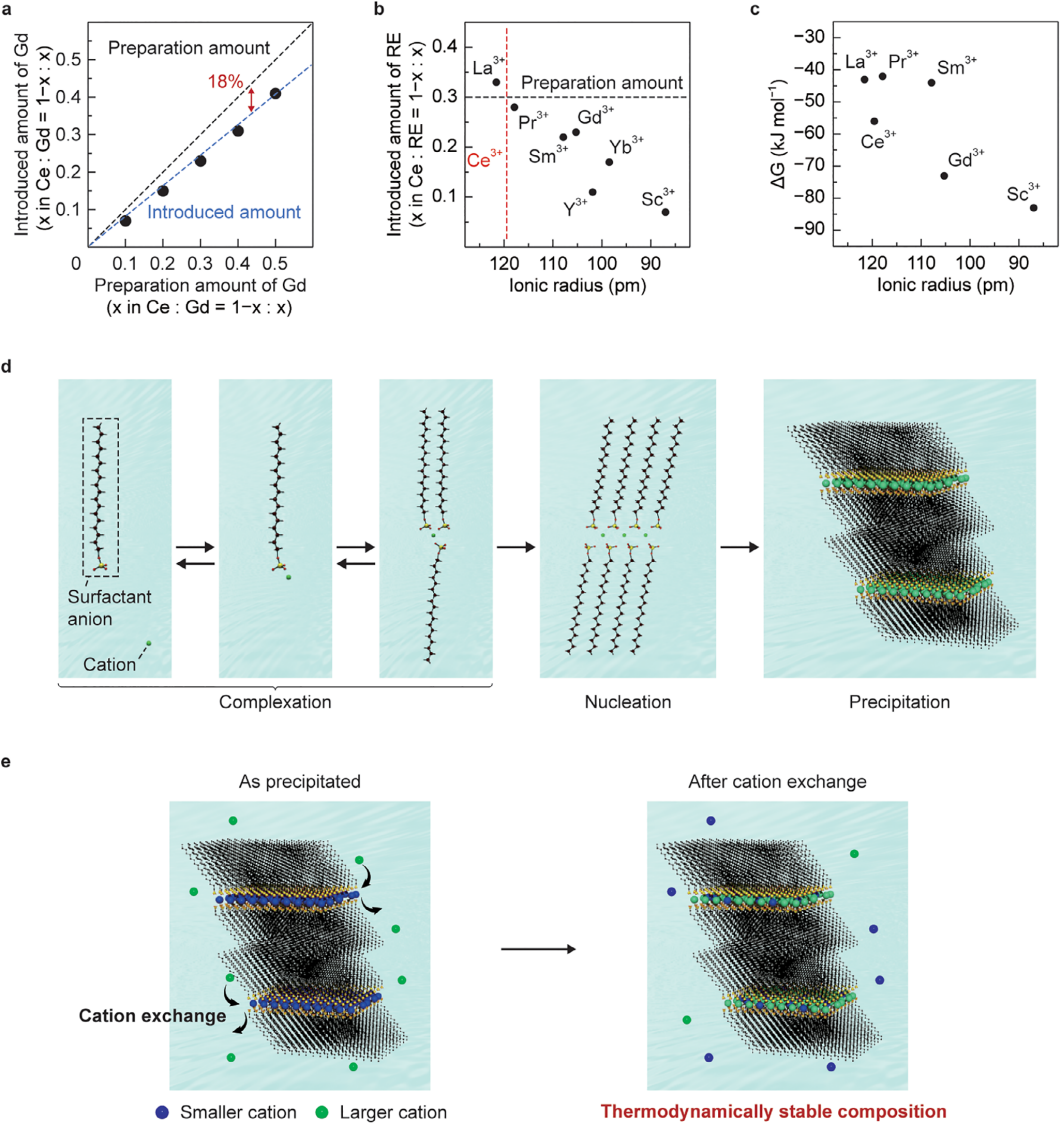
Metal compositions in solid‐state surfactants synthesized with various preparation conditions. (a) Relationship between preparation amount and induced amount of Gd in the solid‐state surfactants investigated by EDXRF. (b) The composition of surfactant crystals synthesized by using Ce and RE (RE = La, Pr, Sm, Gd, Y, Yb, or Sc) with the preparation ratio of Ce:RE = 0.70:0.30 was investigated by EDXRF. (c) The Gibbs free energy changes (ΔG) of the ligand exchange reaction were calculated by DFT calculations. The values of the ionic radii were adopted from the literature [25]. (d) Illustration of the precipitation process of a solid‐state surfactant proposed by Pereira et al. [24]. (e) Illustration of a cation exchange reaction.

We investigated how metal ion incorporation proceeds when varying metal ion concentrations and types to identify the factors affecting the composition of surfactant crystals. Specifically, we used samples synthesized with varying initial preparation ratios of Ce:Gd = 0.90:0.10, 0.80:0.20, 0.60:0.40, and 0.50:0.50. The formation of surfactant crystals was confirmed by XRD and SEM‐EDS, and the amounts of incorporated metal ions in these samples were analyzed using EDXRF, respectively (see supporting information, Figures S3–S5). The resulting compositions were determined to be Ce:Gd = 0.93:0.07 (preparation ratio; Ce:Gd = 0.90:0.10), 0.85:0.15 (0.80:0.20), 0.69:0.31 (0.60:0.40), and 0.59:0.41 (0.50:0.50), as summarized in Figure 2a. These results revealed that the incorporated amounts of Gd decreased by approximately 18% compared to the preparation amounts. This suggests that the metal composition in the solid‐state surfactants can be controlled by adjusting the preparation ratios. In the Gd case, solid‐state surfactants with the desired Ce:Gd compositions can be obtained by setting the preparation ratio to approximately 1.2 times larger than the target value.

To investigate the effect of the type of elements on the preferential incorporation, samples in which Gd was substituted with other rare‐earth (RE) ions (RE = La, Pr, Sm, Yb, Y, or Sc) at a preparation ratio of Ce:RE = 0.70:0.30 were examined. The formation of the surfactant crystals was confirmed by XRD and SEM‐EDS, and the amounts of incorporated metal ions in these samples were analyzed using EDXRF, respectively (see Figures S6–S8). The EDXRF results revealed that the metal compositions were Ce:La = 0.67:0.33, Ce:Pr = 0.72:0.28, Ce:Sm = 0.78:0.22, Ce:Yb = 0.83:0.17, Ce:Y = 0.89:0.11, and Ce:Sc = 0.93:0.07, as shown in Figure 2b. These results demonstrate that the amount of RE incorporated into surfactant crystals depends strongly on the type of rare‐earth cation. A systematic decrease in incorporation was observed with the larger rare‐earth elements being more preferentially incorporated into solid‐state surfactants (La > Pr > Sm ≈ Gd > Yb > Y > Sc) when the incorporated amount of each RE was compared with its ionic radius. To achieve the desired incorporation of metal ions with smaller ionic radii into the surfactant, a larger excess of those metal ions should be supplied.

The origin of the difference between the prepared and incorporated amounts of metal ions was discussed based on the possible formation mechanism of the surfactant crystals and first‐principles calculation using density functional theory (DFT). According to the previous research on surfactant assemblies, the formation mechanism of the solid‐state surfactant crystals was proposed as follows: (1) complexation between metal cations and surfactant anions, (2) nucleation, and (3) precipitation through crystal growth (Figure 2d) [24]. Based on this process, we first hypothesized that cations forming more stable complexes with surfactant anions would be preferentially incorporated into the solid‐state surfactants. To investigate the stability of complexes with surfactant anions and metal cations, the Gibbs free energy changes for the substitution of water molecules in RE (RE = La, Ce, Pr, Sm, Gd, or Sc) aqua complexes with octadecylsulfate anions were evaluated by DFT calculation (Figure S9 and Table S2). The details of the calculations are described in the supporting information. The DFT calculation results showed the values of ΔG = −43 kJ mol^−^^1^ (La), −56 kJ mol^−^^1^ (Ce), −42 kJ mol^−^^1^ (Pr), −73 kJ mol^−^^1^ (Gd), and −83 kJ mol^−^^1^ (Sc), respectively (Figure 2c). These results indicate a trend in which cations with smaller ionic radii exhibit higher reactivity toward ligand exchange with octadecylsulfate anions. This is opposite to the experimental results, suggesting that the critical process determining metal incorporation into the surfactant crystals is not their complexation process.

We therefore considered that the metal composition of the solid‐state surfactant is modified through cation exchange with the surrounding solution after precipitation. Indeed, we experimentally confirmed that such post‐precipitation cation exchange occurs. For example, immersion of a Gd‐containing solid‐state surfactant in an aqueous Ce nitrate solution yielded a mixed Ce–Gd surfactant phase (Figure S10), demonstrating that cation exchange proceeds under these conditions. These results indicate that the final metal composition is determined by thermodynamic stability established through this exchange process.

Considering the alkyl‐chain packing structure, electrostatic repulsion is inherently present between negatively charged headgroups. When a smaller cation with the same valence is introduced, it draws the headgroups closer together, reducing inter‐headgroup distances and increasing electrostatic repulsion. As a result, the structure becomes thermodynamically less stable than that formed with a larger cation (Figure S11). Accordingly, although smaller cations may be initially favored during complexation with surfactant anions, they are subsequently replaced by larger cations through post‐precipitation exchange, leading to their lower incorporation in the final structure.

To examine the generality of this interpretation beyond rare‐earth elements, we investigated Ga^3+^ and Fe^3+^ as non‐rare‐earth trivalent cations in La–Ga and Ga–Fe systems. In both cases, the incorporation order (La > Ga and Fe > Ga; Figure S12) follows the trend in ionic radii based on Shannon effective ionic radii (La^3+^: 116 pm (CN = 8), Fe^3+^ (high‐spin): 64.5 pm (CN = 6), Ga^3+^: 62.0 pm (CN = 6)) [25]. These results support the conclusion that the interaction between metal cations and the geometrically constrained surfactant assembly governs incorporation selectivity. Based on these findings, we propose the following mechanistic picture. First, smaller cations with relatively compact hydration shells preferentially form complexes with surfactant anions. Second, these complexes serve as building units for nucleation and crystal growth, leading to precipitation of the solid‐state surfactant (Figure 2d). Third, post‐precipitation cation exchange with excess cations in solution drives the system toward a thermodynamically more stable composition, resulting in preferential incorporation of larger cations (Figure 2e). This mechanism provides a basis for the rational design of solid‐state surfactants. However, the mechanistic picture proposed here remains a hypothesis derived from experimental observations. Achieving a more rigorous understanding will require a comprehensive theoretical treatment based on advanced methodologies, such as large‐scale simulations that explicitly account for interfaces, solvation effects, and reaction dynamics.

### 2.3 Formation of Rare‐Earth‐Doped Ceria in the Interlayer

**FIGURE 3:**
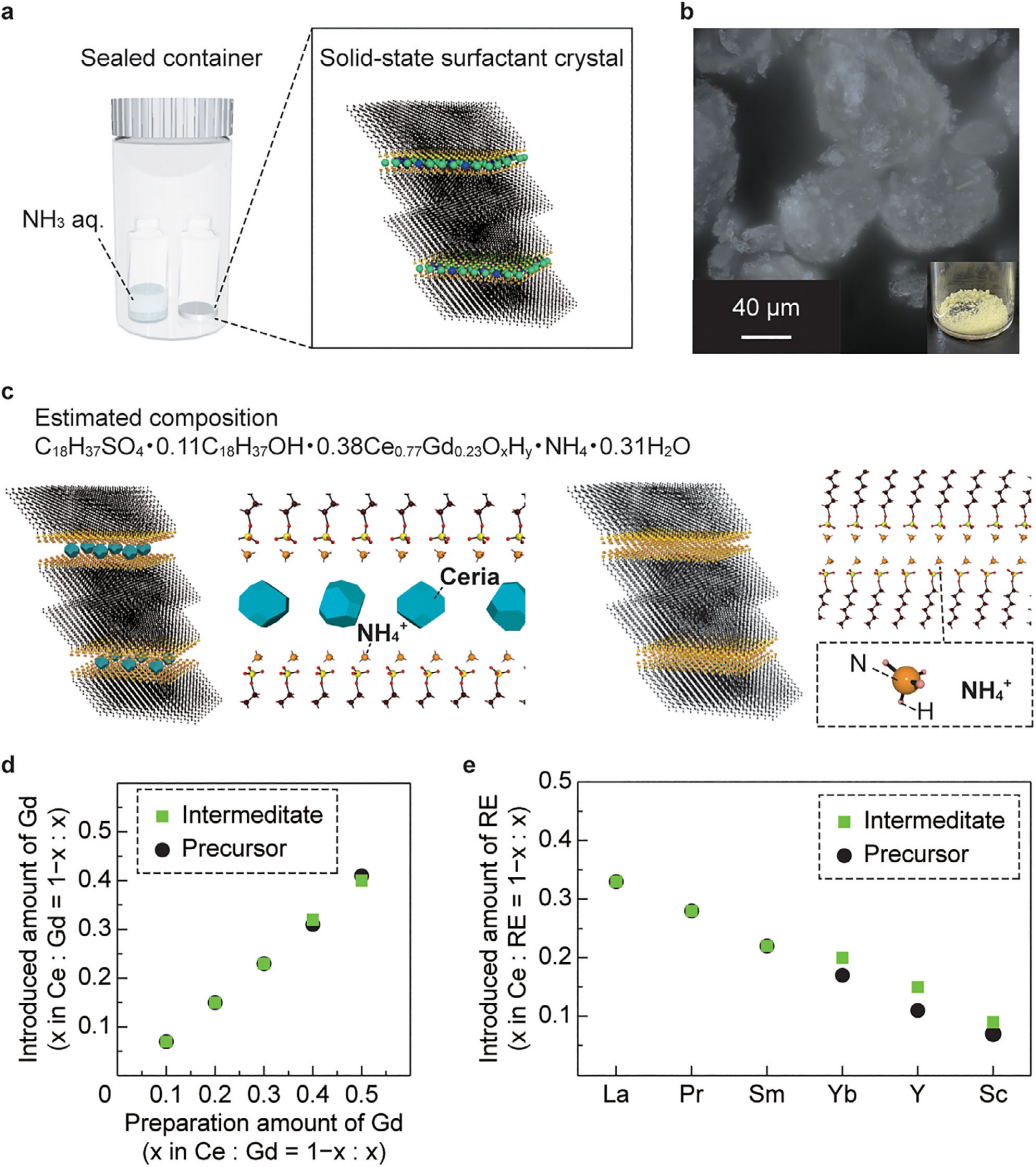
Intermediates obtained by humid ammonia vapor treatment for the solid‐state surfactants. (a) Illustration of humid ammonia vapor treatment. (b) Optical microscope image and (inset) appearance of the crystals after humid ammonia vapor treatment. (c) Estimated composition and predicted structure of intermediate crystal prepared using the solid‐state surfactants synthesized with the preparation ratio of Ce:Gd = 0.70:0.30. (d) The amounts of Gd in the intermediates obtained with various preparation ratios were investigated by EDXRF. (e) The amounts of RE (RE = La, Pr, Sm, Yb, Y, or Sc) in the intermediates obtained with the preparation ratio of Ce:RE = 0.70:0.30 were investigated by EDXRF.

We carried out the humid ammonia vapor treatment to form rare‐earth‐doped ceria using the synthesized solid‐state surfactants as precursors, the surfactant crystals with different molar ratios of Ce:Gd, and the crystals containing both Ce and RE (RE = La, Pr, Sm, Yb, Y, or Sc). The solid‐state surfactants were exposed to humid ammonia vapor at room temperature for two days by placing them in a sealed container alongside an ammonia aqueous solution (Figure 3a). This treatment caused a color change of the precursor powder from white to yellow (Figures 1a and 3b), suggesting the formation of fluorite‐structured ceria with oxygen defects. We denoted the samples after the treatment as intermediate.

The structures and compositions of the intermediates with all preparation ratios were briefly investigated by XRD, EDXRF, and SEM‐EDS. The detailed investigations of the intermediates (TEM, FT‐IR, TG, and CHNS) were conducted for the sample synthesized with the preparation ratio of Ce:Gd = 0.70:0.30 (Figures S13–S21), which indicated the formation of surfactant crystals containing ammonium salt and the crystals containing ceria nanoclusters in the interlayer spaces (the details were discussed in the supporting information, Figure 3c). EDXRF measurement revealed that the metal composition of intermediates almost corresponded to the precursors (Figure 3d,e). This indicates that the metal compositions of intermediates were maintained even after the ammonia vapor treatment process.

### 2.4 Synthesis and Characterization of Rare‐Earth‐Doped Ceria Nanosheets

**FIGURE 4:**
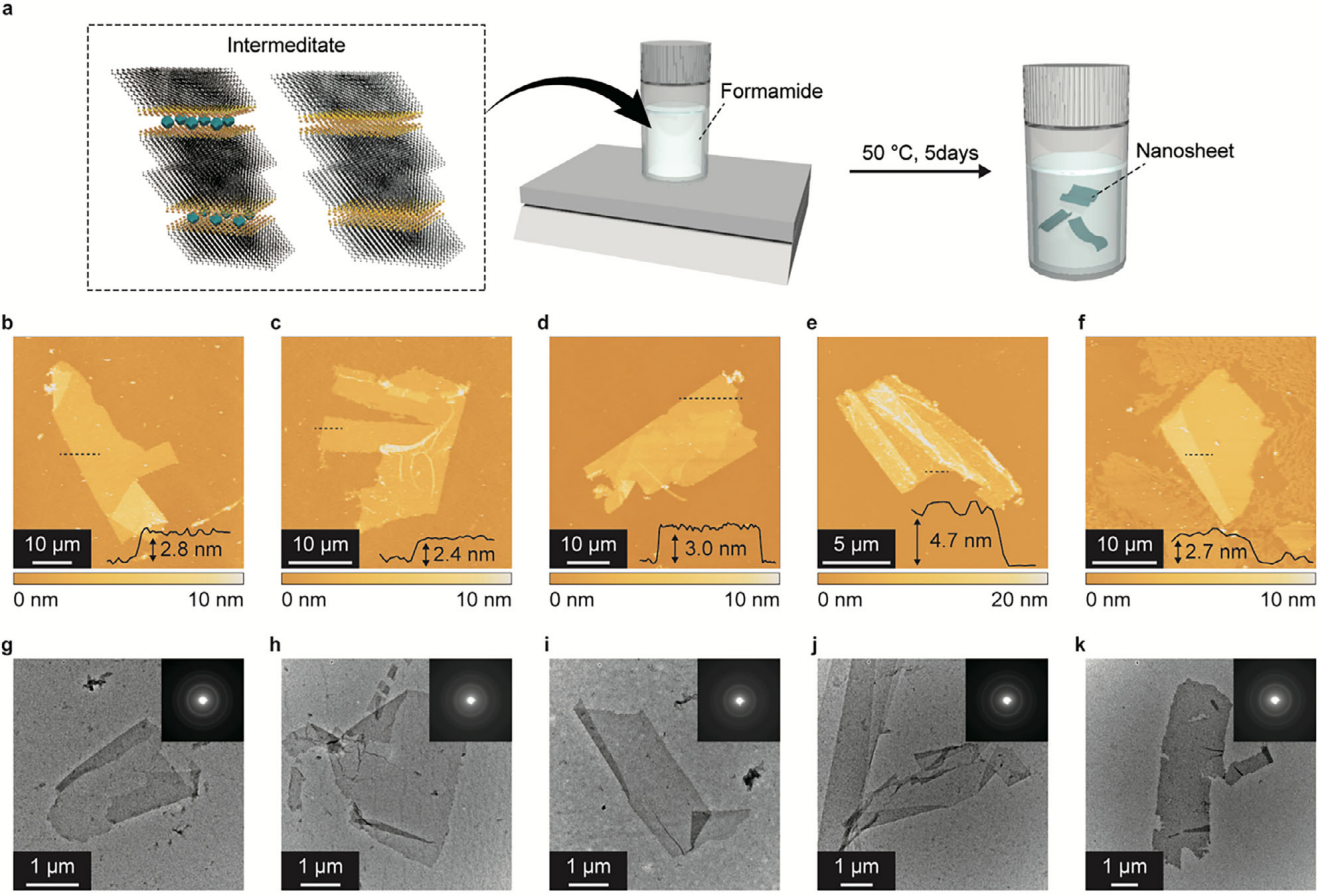
Polycrystalline Gd‐doped ceria nanosheets. (a) Illustration of the preparation process for the nanosheet solution. (b–f) AFM images, and (g–k) TEM image of Gd‐doped ceria nanosheets synthesized with the preparation ratios of Ce:Gd = 0.90:0.10 (b and g), 0.80:0.20 (c and h), 0.70:0.30 (d and i), 0.60:0.40 (e and j), and 0.50:0.50 (f and k).

The obtained intermediates were immersed in formamide and aged at 50°C for five days to obtain a nanosheet solution (Figure 4a). The obtained nanosheets were deposited onto Si substrates and TEM grids and analyzed by atomic force microscopy (AFM), TEM‐EDS, and SEM‐EDS.

AFM results showed that the obtained nanosheets had thicknesses ranging from 2.0 to 4.7 nm and lateral sizes of tens of micrometers (Figure 4b–f and Figure S22). For instance, the nanosheet prepared by using the precursor synthesized with a Ce:Gd preparation ratio of 0.70:0.30 exhibited a thickness of approximately 3 nm and lateral sizes of up to 40 µm. Additionally, some nanosheets showed folded areas, indicating their freestanding nature. Then, the structures and compositions of the nanosheets were analyzed by TEM‐EDS and SEM‐EDS. The selected‐area electron diffraction (SAED) patterns showed ring patterns which almost corresponded to the simulated Debye ring pattern of ceria with a fluorite structure, suggesting the formation of polycrystalline ceria nanosheets (Figure 4g–k and Figures S23–S25). EDS mappings and spectra revealed uniform distributions of Ce and RE elements throughout the nanosheets, confirming their homogeneous incorporation (Figures S23 and S24). Notably, the nanosheets retained their polycrystalline ceria structure and nearly unchanged Gd content even after storage under ambient conditions for more than four months, indicating robust long‐term stability (detailed experiments were shown in Figure S26).

**FIGURE 5:**
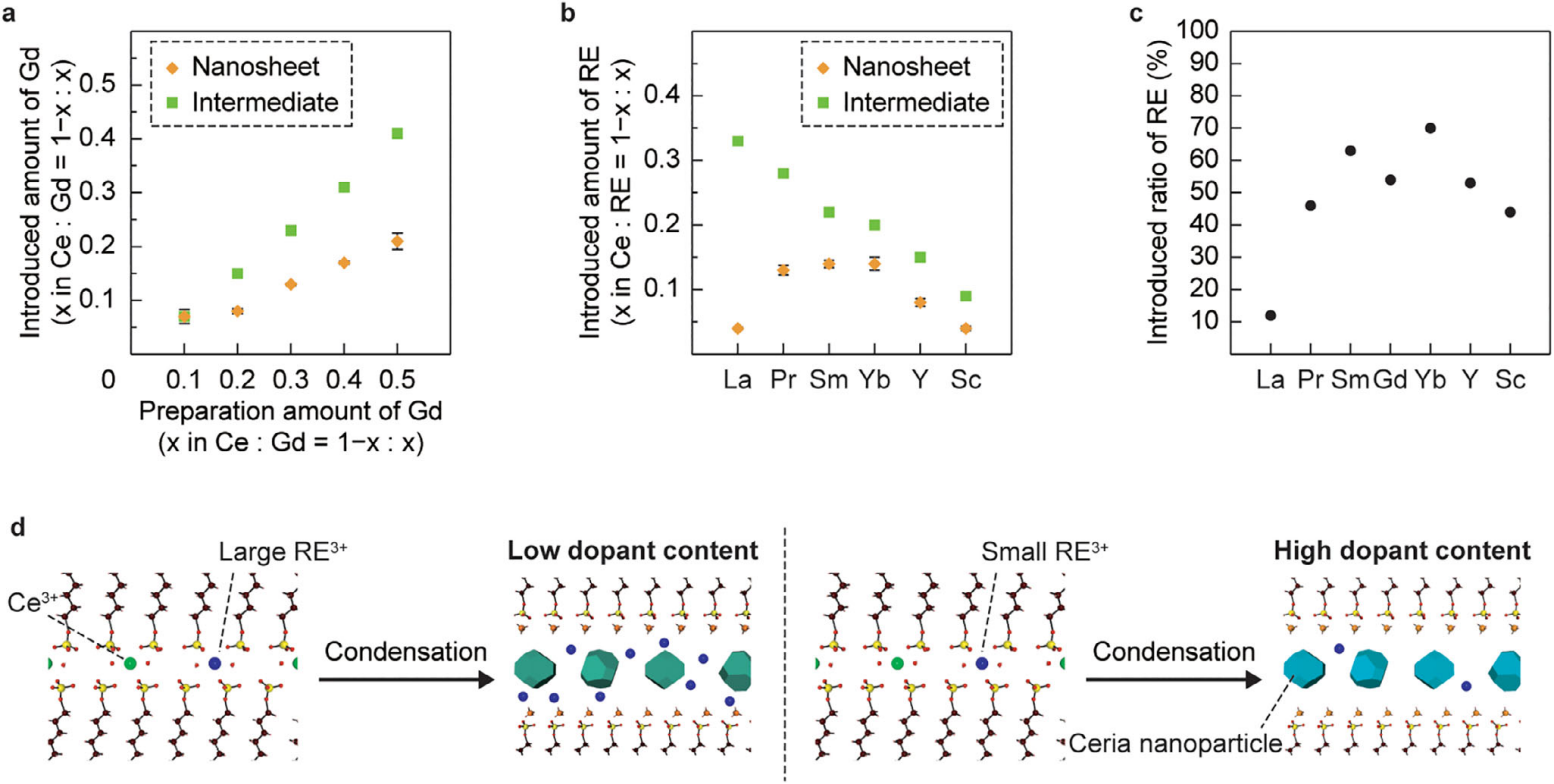
Doping amounts in ceria nanosheets. (a) The amounts of Gd in the nanosheets obtained with various preparation ratios were investigated by SEM‐EDS. (b) The amounts of RE (RE = La, Pr, Sm, Yb, Y, or Sc) in the nanosheets obtained with the preparation ratio of Ce:RE = 0.70:0.30 were investigated by SEM‐EDS. (c) Introduced ratios of RE from intermediates into the ceria nanosheets. (d) Illustration of a possible mechanism for different amounts of RE incorporated into the ceria nanosheets.

The molar ratios of metals incorporated in the nanosheets were investigated by SEM‐EDS (n = 3). The metal compositions of Gd‐doped ceria nanosheets were Ce:Gd = 0.93:0.07 (metal ratio in the intermediates; Ce:Gd = 0.93:0.07), 0.91:0.09 (0.85:0.15), 0.87:0.13 (0.77:0.23), 0.83:0.17 (0.69:0.31), and 0.78:0.22 (0.59:0.41) with corresponding standard deviations of the Gd fractions of 0.001, 0.005, 0.002, 0.003, and 0.015, respectively, as shown in Figure 5a. These data indicated that not all Gd species present in the intermediates were incorporated into the ceria lattice during formation. The incorporated amount in the nanosheets was approximately 54% lower than that in the intermediates. This indicates that approximately 2.2 times the target amount of Gd is required for the initial preparation step to synthesize ceria nanosheets with the desired compositions, considering both the precursor preparation process and the nanosheet formation process.

For other dopants, metal molar ratios were Ce:La = 0.96:0.04 (metal ratio in the intermediates; Ce:La = 0.67:0.33), Ce:Pr = 0.87:0.13 (0.72:0.28), Ce:Sm = 0.86:0.14 (0.78:0.22), Ce:Yb = 0.86:0.14 (0.80:0.20), Ce:Y = 0.92:0.08 (0.85:0.15), and Ce:Sc = 0.96:0.04 (0.91:0.09), with corresponding standard deviations of 0.002, 0.008, 0.006, 0.010, 0.006, and 0.003, respectively, as shown in Figure 5b. The amount of RE elements incorporated into the nanosheets from the intermediates varied depending on the type of RE (La: 12%, Pr: 46%, Sm: 64%, Yb: 82%, Y: 73%, and Sc: 57%, as shown in Figure 5c). This indicates that larger cations are less preferentially incorporated into the nanosheets. This trend can be attributed to differences in reactivity during the condensation of RE elements in the humid ammonia vapor treatment. It is well established that cations with larger ionic radii and lower charge density exhibit lower reactivity in the condensation reaction (Figure 5d) [26]. In fact, computational results demonstrate that larger RE elements possess higher p*K*_a_ values for hydration [27]. Consequently, the incorporation of larger RE cations into ceria nanoparticles during humid ammonia vapor treatment decreases relative to that of smaller RE cations. The pronounced discrepancies among the RE elements may arise from the mild condensation conditions of the humid ammonia vapor treatment. In the synthesis of GaFeO*_x_* nanosheets using Ga and Fe, which are more susceptible to hydrolysis, the Fe incorporation ratio exceeded 90%, providing further support for this interpretation (see the supporting information for details, Figure S27). A more complete understanding of the ammonia vapor reaction will require a comprehensive analysis of the metal species present in the solid‐state surfactant during the reaction, combined with first‐principles calculations. Therefore, it can be concluded that precise doping of nanosheets via the solid‐state surfactant templating method requires control not only over the composition of the precursor surfactant crystals but also over the condensation process.

**FIGURE 6:**
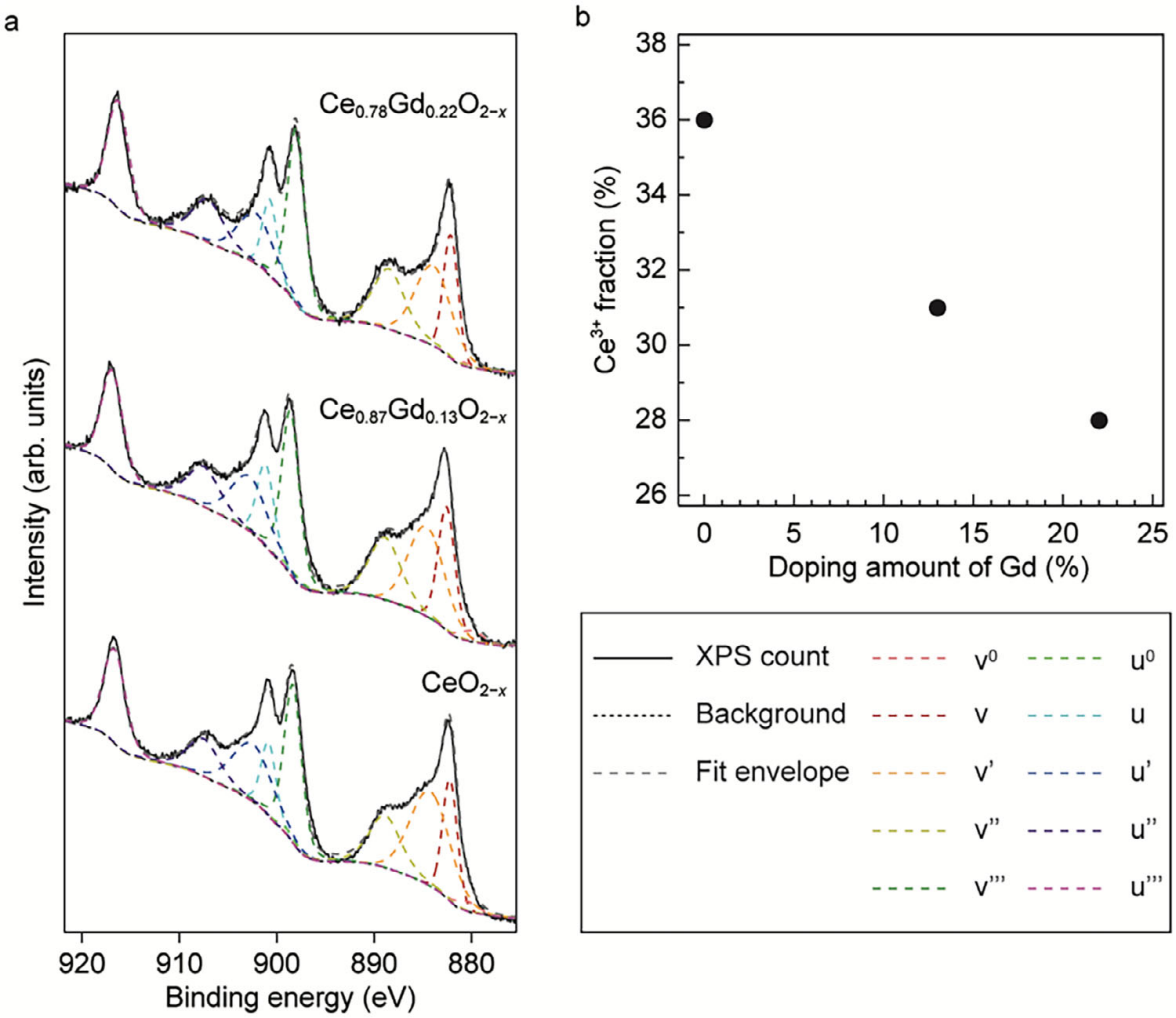
Chemical state of Ce in ceria nanosheets. (a) XPS spectra of ceria nanosheets and (b) Ce^3+^ fractions in the ceria nanosheets obtained by XPS.

Then, the effect of rare‐earth doping on the redox properties of ceria nanosheets was investigated by XPS measurements of CeO_2−_ *_x_*, Ce_0.87_Gd_0.13_O_2−_ *_x_*, and Ce_0.78_Gd_0.22_O_2−_ *_x_* nanosheets (Figure 6a). Because the nanosheets are ultrathin, the XPS signal is considered to reflect an overall chemical state of the nanosheets. Comparison of the spectra revealed that the intensity of the v′ peak at approximately 885 eV, which is attributed to Ce^3+^ species, decreased with increasing Gd content (Figure S28) [22]. To quantify the amount of Ce^3+^ compared to Ce^4+^, the obtained spectra were fitted, and the fraction of Ce^3+^ was calculated from the area ratios of peaks assigned to Ce^3+^ (v^0^, v′, u^0^, and u′) and Ce^4+^ (v, v′″, v′″, u, u′′, and u′′″) [28]. The Ce^3+^ fractions were determined to be 36% for the CeO_2−_ *_x_*, 31% for Ce_0.87_Gd_0.13_O_2−_ *_x_*, and 28% for Ce_0.78_Gd_0.22_O_2−_ *_x_* (Figure 6b). Doping ceria with trivalent metal ions is known to induce oxygen vacancies for charge compensation, which in turn suppresses the reduction of Ce^4+^ accompanied by the formation of oxygen vacancies [29]. This trend is consistent with the established defect chemistry of doped ceria, where trivalent rare‐earth doping introduces oxygen vacancies for charge compensation, thereby reducing the need for vacancy formation via Ce reduction. We consider this as a critical first step, as redox tunability is fundamentally linked to key functionalities such as catalytic activity and ionic transport in ceria‐based systems. In this context, we performed preliminary conductivity measurements using nanosheet‐based devices (Figures S29 and S30). The obtained nanosheets have large lateral dimensions, enabling direct electrode fabrication on individual nanosheets and thereby providing a useful platform for future investigations of their application‐relevant properties. This work provides a robust foundation for systematic investigations, and studies on catalytic performance and ionic transport properties are currently underway.

## 3 Conclusion

We developed a rational strategy for synthesizing solid‐state surfactants with controlled compositions. Compositional analysis of surfactant crystals synthesized using Ce nitrate and Gd nitrate with various preparation ratios indicated that the metal molar ratio in the solid‐state surfactants can be systematically tuned by adjusting the precursor composition. Comparing the compositions of the surfactant crystals synthesized using cerium nitrate and various rare‐earth nitrates (La, Pr, Sm, Gd, Yb, Y, or Sc) revealed a clear preference for incorporation of larger rare‐earth ions into the surfactant lattice, following the trend La > Pr > Sm ≈ Gd > Yb > Y > Sc. Using these compositionally defined surfactants, we synthesized rare‐earth‐doped ceria nanosheets with controlled dopant contents. Although the absolute dopant concentrations in Gd‐doped nanosheets did not exactly match those in the precursor surfactants, a clear proportional relationship was observed. In contrast, the doping amounts of other rare‐earth elements systematically decreased with increasing ionic radius. Notably, Gd incorporation was also found to suppress the reduction of Ce in the ceria nanosheets. These findings provide key design principles for controlling composition and redox behavior in non‐layered 2D oxide nanosheets synthesized via solid‐state surfactant templating, thereby extending this method toward a broadly applicable and predictable synthetic platform.

## Author Contributions

**Kohei Hayashi**: investigation. **Makoto Kobayashi**: writing, review and editing, validation. **Kentaro Ito**: data curation, investigation, validation, formal analysis, visualization, writing – original draft. **Daiki Kurimoto**: investigation. **Eisuke Yamamoto**: conceptualization, methodology, software, data curation, investigation, validation, formal analysis, supervision, funding acquisition, visualization, project administration, resources, writing – review and editing. **Minoru Osada**: supervison, writing – review and editing, funding acquisition.

## Conflicts of Interest

The authors declare no conflicts of interest.

## Supporting information




**Supporting File**: advs75720‐sup‐0001‐SuppMat.docx.

## Data Availability

The data that support the findings of this study are available in the supplementary material of this article.
