# Supplementary material for: Tailored Synthesis of Doped Non‐Layered Oxide Nanosheets Using Designed Solid‐State Surfactants
Source: Adv Sci (Weinh). 2026 May 19;13(45):e75720. doi: 10.1002/advs.75720 (PMC13335775; doi:10.1002/advs.75720)
Supplement: Supplementary file 1 [file ADVS-13-e75720-s001.docx]

**Tailored Synthesis of Doped Non-layered Oxide Nanosheets Using Designed Solid-state Surfactants**

Kentaro Ito, Eisuke Yamamoto,* Kohei Hayashi, Daiki Kurimoto, Makoto Kobayashi, and Minoru Osada*

**Table of Contents**

**Section A. Materials and Characterization**

**Section B. Experimental section**

**Section C. Supplementary figures and tables**

**Section A. Materials and Characterization**

**Materials.** Cerium(Ⅲ) nitrate hexahydrate (98.0+%), gadolinium nitrate hexahydrate (99.5%), samarium nitrate hexahydrate (99.5%), ytterbium nitrate n-hydrate (99.9%), iron(III) chloride hexahydrate (99.0%), and formamide (98.5%) were purchased from Fujifilm Wako Pure Chemicals Co. The hydration number of ytterbium nitrate was determined to be 7.6 by TG-DTA measurement. Praseodymium(Ⅲ) nitrate hexahydrate (99.95%) was purchased from Kanto Chemical Co., Inc. Yttrium nitrate hexahydrate (99.9%) was purchased from Thermo Fisher Scientific Inc. Scandium nitrate tetrahydrate (99.9%) was purchased from Mitsuwa Chemicals Co., Ltd. Sodium octadecylsulfate (93%) was purchased from Sigma-Aldrich. Ammonia solution (28%) was purchased from Kishida Chemical Co., Ltd. Gallium nitrate hydrate was purchased from Kojundo Chemical Laboratory Co., Ltd. All the materials were used without further purification.

**Characterization.** X-ray diffraction (XRD) patterns were recorded using a SmartLab diffractometer (Rigaku) operated at 45 kV and 200 mA. Scanning electron microscopy (SEM) images were obtained with a JSM-7610F Plus (JEOL). Transmission electron microscopy (TEM) images and selected-area electron diffraction (SAED) patterns were acquired using a JEM-2100F/HK (JEOL). Atomic force microscopy (AFM) measurements were performed with an MFP-3D system (Oxford Instruments). Prior to microscopic analyses, organic components were removed by heating the samples at 500 °C for 6 h. Confocal laser microscopy images were obtained using an OLS4000 (Olympus). Carbon, hydrogen, nitrogen, and sulfur (CHNS) elemental analyses were carried out with a 2400 II CHNS/O analyzer (PerkinElmer). Thermogravimetric (TG) measurements were performed on a STA7200 (Hitachi) under air flow at a heating rate of 10 °C min⁻^1^ up to 1000 °C. Energy-dispersive X-ray fluorescence (EDXRF) measurements were conducted using a JSX-1000S spectrometer (JEOL). Inductively coupled plasma-atomic emission spectroscopy (ICP-AES) measurements were carried out with a SPS7800 (Seiko Instruments). X-ray photoelectron spectroscopy (XPS) measurements were performed with an ESCALAB 250Xi (Thermo Fisher Scientific) for precursor solid-state surfactants and a PHI 5000 VersaProbe III (ULVAC-PHI) for nanosheets. Quantitative analysis of the Ce^3^⁺ fraction was carried out by peak fitting using MultiPak software (ULVAC-PHI), with background subtraction performed using the Smart method.

**Section B. Experimental section**

**Synthesis of solid-state surfactant crystals.** Sodium octadecyl sulfate (100 mg) was dissolved in 15 mL of water by heating and stirring at 70 °C. Separately, metal nitrates were dissolved in 10 mL of water by heating and stirring at 70 °C. The amounts of metal nitrates were adjusted such that the molar quantity of metal ions was 14 times that of the surfactant. The metal nitrate solution was then added to the surfactant solution, resulting in the formation of a white precipitate. After stirring for 30 seconds, the mixture was left to stand at room temperature overnight. The precipitate was subsequently collected by filtration and thoroughly washed with water. Following washing, the precipitate was air-dried for one day and then dried by heating at 60 °C.

**Theoretical calculation.** All calculations were performed using Gaussian 16.^[1]^ We performed the geometry optimizations for aqua complexes of rare-earth elements (La, Pr, Ce, Sm, Gd, and Sc) and for the complexes in which one aqua ligand was substituted with an octadecyl sulfate anion. Then, we calculated the Gibbs free energy changes in the ligand exchange reactions shown in Eq. (1), including thermal correction.

$$\begin{aligned} \left[ Me\left( H_{2}O \right)_{n} \right]^{3+}+{{ODSO}_{4}}^{-}\to\left[ Me\left( H_{2}O \right)_{n-1}\left( {ODSO}_{4} \right) \right]^{2+}+H_{2}O \#\left( 1 \right) \end{aligned}$$

where *Me* denotes a metal and *ODSO_4_*^-^ denotes the octadecylsulfate anion. The geometry optimizations were conducted at the ωB97X-D/def2-TZVP level.^[2,3]^ For lanthanoids, the corresponding def2-ECP was employed. The coordination numbers of complexes were determined based on previous reports.^[4,5]^ The convergence criteria for SCF calculations and structural optimizations were set to the default values in Gaussian16: for SCF calculations, the energy threshold was 1.00 × 10⁻^6^ Hartree; for structural optimizations, the criteria were Maximum force ≤ 0.000450 Hartree Bohr⁻^1^, RMS force ≤ 0.000300 Hartree Bohr⁻^1^, Maximum displacement ≤ 0.001800 Å, and RMS displacement ≤ 0.001200 Å. We performed the frequency calculations for the optimized structures and confirmed that no imaginary frequencies were found. Solvent effects were included using the polarizable continuum model (PCM) with water as the solvent.^[6,7]^ Thermal corrections at 298.15 K were applied to the energy of each molecule. The optimized structures are shown in Figure S9 and Table S2.

**Humid ammonia vapor treatment.** Solid-state surfactant crystals (30 mg) were put into a 3 mL screw-cap vial (Nichiden-Rika Glass Co.). Separately, 2 mL of a 28% ammonia solution was placed into another 3 mL screw-cap vial. Both vials were positioned uncapped inside a 50 mL screw-cap vial (Nichiden-Rika Glass Co.), which was subsequently sealed with a gasket-equipped cap. The sealed container was left to stand at 25 °C for two days. Then, the resulting powder was collected by filtration and thoroughly washed with water. The obtained powder was air-dried for one day.

**Preparation of nanosheet solution.** Intermediate powder (10 mg) was dispersed in 10 mL of formamide. The mixture was heated on a hotplate set at 50 °C for five days, resulting in a nanosheet colloidal solution. The nanosheets were transferred onto Si substrates by drop-casting and onto TEM grids by dipping.

**Section C. Supplementary figures and tables**

**Figure S1.** Characterization of the solid-state surfactants synthesized by using both Ce and Gd with the preparation ratio of Ce:Gd = 0.70:0.30. (a) TEM image, (b) EDXRF spectrum, (c) SEM image, (d) corresponding EDS spectrum, and EDS mappings of (e) Ce L, (f) Gd L, and (g) S K of the solid-state surfactants synthesized with the preparation ratio of Ce:Gd = 0.70:0.30.

**Figure S2.** Characterization of the solid-state surfactants synthesized by using both Ce and Gd with the preparation ratio of Ce:Gd = 0.70:0.30. XPS spectra of (a) Ce 3d core level, (b) Gd 4d core level, and (c) S 2p core level, (d) FT-IR spectrum, and (e) TG curve of solid-state surfactants synthesized with the preparation ratio of Ce:Gd = 0.70:0.30.

**Table S1.** Composition of the surfactant crystals.

| Sample | Me (wt.%)  (Investigated by the amount of residue after TG measurement) | C (wt.%)  (Investigated by CHNS analysis) | H(wt.%) (Investigated by CHNS analysis) | N (wt.%) (Investigated by CHNS analysis) | S(wt.%) (Investigated by CHNS analysis) | Estimated composition  (Amount of H_2_O estimated by TG curve) | O (wt.%)  (Estimated by predicted composition) | Total (wt.%) |
| --- | --- | --- | --- | --- | --- | --- | --- | --- |
| Reagent surfactant crystals (Na) | - | 58.01 | 10.38 | Neg. | 7.43 | 0.15 C_18_H_37_OH⦁  C_18_H_37_SO_4_⦁  n H_2_O^[a]^ | - | - |
| Precursor surfactant crystals | 11.54 | 54.43 | 9.57 | Neg. | 7.34 | 0.10C_18_H_37_OH⦁  C_18_H_37_SO_4_⦁  0.32(0.77Ce⦁0.23Gd)⦁  0.58H_2_O | 17.2^[b]^ | 100 |
| Intermediate surfactant crystals | 12.35 | 53.96 | 10.61 | 1.79 | 3.93 | 0.11C_18_H_37_OH⦁  C_18_H_37_SO_4_⦁  0.38(0.77Ce⦁0.23Gd)O_x_H_y_⦁  NH_4_⦁  0.31 H_2_O | 15.2^[b]^ | 98.2 |

^[a]^ The existence of octadecanol was clearly detected by ^1^H nuclear magnetic resonance (NMR) and ^13^C cross-polarization magic-angle-spinning (CP-MAS) NMR spectra, despite the high-purity inspection certificate (<97%) obtained by the reagent company using high-performance liquid chromatography (HPLC). ^[b]^ The amount was calculated with the assumption that the residues were obtained as Ce_0.77_Gd_0.23_O_1.89_.

**<Preparation of the surfactant crystals with different Gd amounts>**

The formation of surfactant crystals was confirmed by structural analysis using XRD (Figure S3) and compositional analysis via SEM-EDS (Figure S4) and EDXRF (Figure S5). The XRD results revealed the formation of a lamellar structure characteristic of the *L*_c_ phase, and SEM-EDS mappings showed the homogeneous distribution of Ce, Gd, and S within the crystals. For samples prepared with varying Ce:Gd molar ratios, the resulting compositions were determined to be Ce:Gd = 0.93:0.07 (preparation ratio; Ce:Gd = 0.90:0.10), 0.85:0.15 (0.80:0.20), 0.69:0.31 (0.60:0.40), and 0.59:0.41 (0.50:0.50) by EDXRF, as summarized in Figure 2a.

**Figure S3.** XRD patterns of surfactant crystals synthesized by using Ce and Gd with the various preparation ratios of Ce:Gd = 0.90:0.10, 0.80:0.20, 0.60:0.40, and 0.50:0.50.

**Figure S4.** SEM images, EDS mappings, and EDS spectra of surfactant crystals synthesized by using Ce and Gd with the various preparation ratios of Ce:Gd = 0.90:0.10, 0.80:0.20, 0.60:0.40, and 0.50:0.50.

**Figure S5.** EDXRF spectra of surfactant crystals synthesized by using Ce and Gd with the various preparation ratios of Ce:Gd = 0.90:0.10, 0.80:0.20, 0.60:0.40, and 0.50:0.50.

**<Preparation of the surfactant crystals with different RE>**

The formation of surfactant crystals was confirmed by structural analysis using XRD (Figure S6) and compositional analysis by SEM-EDS (Figure S7) and EDXRF (Figure S8). The XRD results revealed the formation of a lamellar structure characteristic of the *L*_c_ phase. In the low-angle region, multiple sets of periodic diffraction peaks were observed. However, SEM-EDS measurements revealed that the metal elements were uniformly distributed in all crystals examined, indicating that the metal species are homogeneously incorporated even in crystals exhibiting different periodic structures. The differences in periodicity are therefore attributed to variations in the amount of water incorporated into the lamellar interlayers during the formation of the surfactant crystals.^[8]^ Subsequently, the metal compositions of the obtained surfactant crystals were quantitatively analyzed using EDXRF. The EDXRF spectra displayed distinct peaks corresponding to Ce Lα (4.8 keV), as well as fingerprint peaks of other rare-earth elements: La Lα (4.7 keV), Pr Lα (5.0 keV), Sm Lβ1 (6.2 keV), Gd Lβ1 (6.7 keV), and Yb Lα (7.4 keV). The metal compositions of the surfactants were Ce:La = 0.67:0.33, Ce:Pr = 0.72:0.28, Ce:Sm = 0.78:0.22, and Ce:Yb = 0.83:0.17, respectively, as shown in Figure 2b.

**Figure S6.** XRD patterns of surfactant crystals synthesized by using Ce and RE (RE = La, Pr, Sm, Yb, Y, or Sc) with the preparation ratio of Ce:RE = 0.70:0.30.

**Figure S7.** SEM images, EDS mappings, and EDS spectra of surfactant crystals synthesized by using Ce and RE (RE = La, Pr, Sm, Yb, Y, or Sc) with the preparation ratio of Ce:RE = 0.70:0.30.

**Figure S8.** EDXRF spectra of surfactant crystals synthesized by using Ce and RE (RE = La, Pr, Sm, Yb, Y, or Sc) with the preparation ratio of Ce:RE = 0.70:0.30.

**Figure S9.** Illustration of optimized structures of RE (RE = La, Ce, Pr, Sm, Gd, and Sc) complexes.

**Table S2.** Optimized structures of RE (RE = La, Ce, Pr, Sm, Gd, and Sc) complexes.

[La(H_2_O)_9_]^3+^

| Atom | Coordinate (Å) | | | Atom | Coordinate (Å) | | |
| --- | --- | --- | --- | --- | --- | --- | --- |
|  | X | Y | Z |  | X | Y | Z |
| O | -1.85364 | -1.82246 | -0.48113 | H | 2.73815 | 0.49631 | -2.43463 |
| O | -1.13698 | 0.48985 | -2.4643 | H | 0.84929 | -1.99592 | -3.21822 |
| H | -2.01948 | -2.48148 | 0.19798 | H | -0.25554 | -2.8285 | -2.51116 |
| H | -2.68191 | -1.66109 | -0.93907 | H | -0.26151 | -1.96264 | 2.33706 |
| O | 1.78052 | 0.42518 | -2.44008 | H | 0.82687 | -2.8106 | 1.62614 |
| O | 0.31547 | -2.06164 | -2.42262 | H | 2.01836 | -0.09933 | 2.30632 |
| O | 0.2093 | -2.08454 | 1.50894 | H | 2.16952 | 1.28509 | 1.62425 |
| O | 1.77913 | 0.41266 | 1.53023 | H | 0.8071 | 2.81564 | -0.98165 |
| O | 0.36347 | 2.24028 | -0.35352 | H | -0.26407 | 2.78016 | 0.13369 |
| O | -1.17862 | 0.46239 | 1.54176 | H | -2.13746 | 0.51019 | 1.56571 |
| O | 2.43363 | -1.80117 | -0.40834 | H | -0.84694 | 0.91992 | 2.31842 |
| H | -1.38763 | -0.07167 | -3.20229 | H | 3.24342 | -1.59279 | 0.06397 |
| H | -1.69174 | 1.27305 | -2.4973 | H | 2.64662 | -2.46224 | -1.07157 |
| H | 1.45645 | 0.94205 | -3.18218 | La | 0.28239 | -0.34804 | -0.45136 |

[La(H_2_O)_8_(ODSO_4_)]^2+^

| Atom | Coordinate (Å) | | | Atom | Coordinate (Å) | | |
| --- | --- | --- | --- | --- | --- | --- | --- |
|  | X | Y | Z |  | X | Y | Z |
| S | -0.33055 | 0.79588 | -0.18491 | H | 2.83625 | -4.58164 | 4.45406 |
| O | -1.40374 | 1.76532 | 0.06897 | H | 1.79324 | -5.58977 | 3.47032 |
| O | -0.71173 | -0.18771 | -1.17979 | C | 1.452 | -5.77922 | 5.58243 |
| O | 0.95097 | 1.3963 | -0.39035 | H | 0.4218 | -6.14003 | 5.48644 |
| O | -4.83857 | -0.39694 | 1.8532 | H | 1.47241 | -5.1421 | 6.47361 |
| O | -5.814 | 0.94137 | -0.94227 | C | 2.38255 | -6.96734 | 5.78928 |
| H | -4.44037 | -0.6172 | 2.70072 | H | 3.41117 | -6.60581 | 5.89861 |
| H | -5.74379 | -0.71662 | 1.86336 | H | 2.37226 | -7.59226 | 4.88914 |
| O | -3.54691 | 2.45854 | -1.8471 | C | 2.01984 | -7.82393 | 6.99545 |
| O | -2.49741 | 0.90616 | 2.7761 | H | 0.98975 | -8.18172 | 6.88701 |
| O | -2.66048 | 3.78636 | 1.71588 | H | 2.0356 | -7.20414 | 7.89895 |
| O | -5.06738 | 3.91644 | 0.07362 | C | 2.94767 | -9.01694 | 7.186 |
| O | -4.92143 | 2.61168 | 2.84747 | H | 3.97656 | -8.65903 | 7.30468 |
| H | -5.76208 | 0.09299 | -1.391 | H | 2.93949 | -9.62755 | 6.27606 |
| H | -6.74154 | 1.18272 | -0.88657 | C | 2.57919 | -9.89126 | 8.37763 |
| H | -4.11717 | 2.08122 | -2.52245 | H | 1.54917 | -10.24626 | 8.25973 |
| H | -2.66872 | 2.54121 | -2.2281 | H | 2.5916 | -9.28456 | 9.29006 |
| H | -2.43096 | 1.44555 | 3.56719 | C | 3.50478 | -11.0879 | 8.55595 |
| H | -1.60464 | 0.60764 | 2.54504 | H | 4.53396 | -10.73298 | 8.68108 |
| H | -1.69966 | 3.76048 | 1.67946 | H | 3.4977 | -11.68816 | 7.63914 |
| H | -2.92572 | 4.63793 | 1.35542 | C | 3.13205 | -11.97439 | 9.73722 |
| H | -5.07188 | 4.22343 | -0.83737 | H | 2.1022 | -12.32753 | 9.6125 |
| H | -5.8525 | 4.26524 | 0.50283 | H | 3.14175 | -11.37671 | 10.65562 |
| H | -5.71458 | 2.34315 | 3.31591 | C | 4.0563 | -13.17324 | 9.90758 |
| H | -4.56213 | 3.38188 | 3.29481 | H | 5.08559 | -12.82017 | 10.03689 |
| La | -3.86763 | 1.68281 | 0.64263 | H | 4.05001 | -13.76688 | 8.98648 |
| O | -0.30215 | 0.03731 | 1.21918 | C | 3.68078 | -14.06723 | 11.0823 |
| C | 0.636 | -1.05155 | 1.3779 | H | 2.65127 | -14.41969 | 10.95297 |
| H | 1.64268 | -0.63287 | 1.42327 | H | 3.68807 | -13.47494 | 12.00422 |
| H | 0.55699 | -1.70641 | 0.50836 | C | 4.60472 | -15.26692 | 11.24859 |
| C | 0.28166 | -1.79038 | 2.64649 | H | 5.63391 | -14.91441 | 11.38038 |
| H | 0.32792 | -1.09988 | 3.49335 | H | 4.5993 | -15.85708 | 10.32525 |
| H | -0.74862 | -2.14936 | 2.57273 | C | 4.22782 | -16.16482 | 12.41977 |
| O | -3.37859 | -0.47626 | -0.74932 | H | 3.19874 | -16.51814 | 12.28814 |
| H | -3.5733 | -1.30433 | -0.30108 | H | 4.23303 | -15.57564 | 13.34388 |
| H | -2.44329 | -0.5259 | -1.04036 | C | 5.15205 | -17.36499 | 12.58558 |
| C | 1.22381 | -2.96482 | 2.88379 | H | 6.17939 | -17.01083 | 12.71783 |
| H | 2.25165 | -2.5968 | 2.96495 | H | 5.14677 | -17.9519 | 11.66153 |
| H | 1.20012 | -3.62919 | 2.01379 | C | 4.76545 | -18.25693 | 13.75794 |
| C | 0.87416 | -3.76484 | 4.132 | H | 3.75272 | -18.64808 | 13.63335 |
| H | -0.15625 | -4.1275 | 4.05082 | H | 5.44291 | -19.10729 | 13.85385 |
| H | 0.90176 | -3.10582 | 5.00639 | H | 4.7922 | -17.69984 | 14.69767 |
| C | 1.80782 | -4.94684 | 4.35745 |  |  |  |  |

[Ce(H_2_O)_9_]^3+^

| Atom | Coordinate (Å) | | | Atom | Coordinate (Å) | | |
| --- | --- | --- | --- | --- | --- | --- | --- |
|  | X | Y | Z |  | X | Y | Z |
| Ce | 0 | 0 | -0.00838 | H | 0.7673 | 1.88303 | 2.49553 |
| O | 0 | 0 | -2.55179 | H | 2.07718 | 1.80622 | 1.66839 |
| O | 0 | 2.4116 | -0.82734 | H | 3.1134 | 0.09417 | -0.65314 |
| H | 0.77457 | 0.02617 | -3.11952 | H | 2.60658 | 1.399 | -1.32375 |
| H | -0.77457 | -0.02617 | -3.11952 | H | 0.14724 | -2.68861 | -1.7353 |
| O | 1.15621 | 1.53439 | 1.68956 | H | -0.37103 | -3.15802 | -0.34941 |
| O | 2.33336 | 0.63109 | -0.81577 | H | -0.7673 | -1.88303 | 2.49553 |
| O | 0 | -2.4116 | -0.82734 | H | -2.07718 | -1.80622 | 1.66839 |
| O | -1.15621 | -1.53439 | 1.68956 | H | -2.2104 | 1.07499 | 2.05603 |
| O | -1.77099 | 1.3393 | 1.24367 | H | -1.97561 | 2.26536 | 1.09033 |
| O | -2.33336 | -0.63109 | -0.81577 | H | -2.60658 | -1.399 | -1.32375 |
| O | 1.77099 | -1.3393 | 1.24367 | H | -3.1134 | -0.09417 | -0.65314 |
| H | -0.14724 | 2.68861 | -1.7353 | H | 2.2104 | -1.07499 | 2.05603 |
| H | 0.37103 | 3.15802 | -0.34941 | H | 1.97561 | -2.26536 | 1.09033 |

[Ce(H_2_O)_8_(ODSO_4_)]^2+^

| Atom | Coordinate (Å) | | | Atom | Coordinate (Å) | | |
| --- | --- | --- | --- | --- | --- | --- | --- |
|  | X | Y | Z |  | X | Y | Z |
| S | 13.8601 | -4.33401 | 7.3362 | H | 15.41514 | -11.20192 | 10.611 |
| O | 12.82494 | -3.34525 | 7.67144 | C | 15.41621 | -11.27249 | 12.76046 |
| O | 13.52022 | -5.09557 | 6.1515 | H | 14.35348 | -11.52166 | 12.85625 |
| O | 15.18622 | -3.80049 | 7.35376 | H | 15.65085 | -10.60358 | 13.59604 |
| O | 8.95764 | -5.32622 | 8.29 | C | 16.24478 | -12.54426 | 12.88805 |
| O | 8.53811 | -3.31364 | 6.11214 | H | 17.30592 | -12.29589 | 12.77361 |
| H | 9.14161 | -5.89977 | 9.03931 | H | 15.99797 | -13.22072 | 12.06211 |
| H | 8.03929 | -5.45608 | 8.04026 | C | 16.04093 | -13.27043 | 14.21146 |
| O | 11.0958 | -1.96448 | 5.78906 | H | 14.97973 | -13.51666 | 14.32988 |
| O | 11.29839 | -4.64332 | 9.85578 | H | 16.29222 | -12.59385 | 15.03603 |
| O | 11.43087 | -1.59243 | 9.63119 | C | 16.86924 | -14.54265 | 14.33654 |
| O | 9.27475 | -0.92765 | 7.79443 | H | 17.92896 | -14.29735 | 14.20353 |
| O | 8.91948 | -2.80361 | 9.99218 | H | 16.60837 | -15.22533 | 13.52002 |
| H | 8.51554 | -3.97737 | 5.41807 | C | 16.68448 | -15.25805 | 15.66861 |
| H | 7.70902 | -2.83063 | 6.08247 | H | 15.62475 | -15.50149 | 15.80479 |
| H | 10.59916 | -2.0822 | 4.97486 | H | 16.94905 | -14.57545 | 16.48404 |
| H | 12.02531 | -1.91169 | 5.55093 | C | 17.51224 | -16.53085 | 15.79153 |
| H | 11.1835 | -4.4789 | 10.79411 | H | 18.57074 | -16.28824 | 15.64463 |
| H | 12.19936 | -4.97376 | 9.71462 | H | 17.24061 | -17.21789 | 14.9822 |
| H | 12.34646 | -1.73242 | 9.88914 | C | 17.34153 | -17.23821 | 17.12976 |
| H | 11.32818 | -0.64932 | 9.47303 | H | 16.28297 | -17.47904 | 17.27909 |
| H | 9.34947 | -0.33129 | 7.04487 | H | 17.61624 | -16.55144 | 17.9383 |
| H | 8.51008 | -0.6602 | 8.31077 | C | 18.16829 | -18.51184 | 17.25076 |
| H | 8.18697 | -3.32948 | 10.32163 | H | 19.22597 | -18.27166 | 17.09422 |
| H | 9.26711 | -2.29259 | 10.72837 | H | 17.8888 | -19.20152 | 16.44636 |
| O | 13.70882 | -5.32083 | 8.58056 | C | 18.00704 | -19.21388 | 18.59297 |
| C | 14.54286 | -6.5021 | 8.61534 | H | 16.94927 | -19.45241 | 18.75128 |
| H | 15.58738 | -6.18798 | 8.57844 | H | 18.28903 | -18.52462 | 19.39686 |
| H | 14.31686 | -7.10954 | 7.73733 | C | 18.83248 | -20.48856 | 18.71231 |
| C | 14.23667 | -7.24364 | 9.89472 | H | 19.88973 | -20.25042 | 18.54981 |
| H | 14.4329 | -6.58456 | 10.74475 | H | 18.54766 | -21.17939 | 17.91075 |
| H | 13.17331 | -7.49864 | 9.91671 | C | 18.67676 | -21.18771 | 20.05656 |
| O | 10.79718 | -4.9979 | 6.08249 | H | 17.61946 | -21.42505 | 20.22073 |
| H | 10.42006 | -5.85925 | 6.28485 | H | 18.96339 | -20.49777 | 20.85843 |
| H | 11.75494 | -5.14009 | 5.93044 | C | 19.50093 | -22.46387 | 20.17557 |
| C | 15.07384 | -8.51132 | 10.01903 | H | 20.55649 | -22.22577 | 20.0098 |
| H | 16.136 | -8.25302 | 9.95778 | H | 19.21273 | -23.15273 | 19.37509 |
| H | 14.86507 | -9.17066 | 9.1704 | C | 19.3397 | -23.1532 | 21.52402 |
| C | 14.81499 | -9.26275 | 11.31855 | H | 18.29665 | -23.42765 | 21.69978 |
| H | 13.75117 | -9.51478 | 11.38609 | H | 19.9394 | -24.06326 | 21.58226 |
| H | 15.02988 | -8.60204 | 12.16534 | H | 19.65072 | -22.49452 | 22.33862 |
| C | 15.64444 | -10.53348 | 11.44825 | Ce | 10.37665 | -3.22538 | 7.91319 |
| H | 16.70677 | -10.28119 | 11.35728 |  |  |  |  |

[Pr(H_2_O)_9_]^3+^

| Atom | Coordinate (Å) | | | Atom | Coordinate (Å) | | |
| --- | --- | --- | --- | --- | --- | --- | --- |
|  | X | Y | Z |  | X | Y | Z |
| O | -1.91841 | -1.57984 | -0.48793 | H | 1.92838 | -0.09498 | -3.13666 |
| O | -1.11897 | 0.42291 | -2.37093 | H | 0.78108 | -2.88446 | -2.36238 |
| H | -2.13002 | -2.402 | -0.93733 | H | -0.25382 | -1.99229 | -3.09717 |
| H | -2.61008 | -1.41254 | 0.15825 | H | -0.25348 | -2.9071 | 1.46418 |
| O | 1.81358 | 0.40328 | -2.32302 | H | 0.81732 | -2.07399 | 2.21625 |
| O | 0.35182 | -2.0249 | -2.35161 | H | 2.65083 | 0.31261 | 1.5494 |
| O | 0.26271 | -2.09772 | 1.43193 | H | 1.43102 | 0.95617 | 2.26351 |
| O | 1.69971 | 0.44056 | 1.49923 | H | 0.87693 | 2.70287 | 0.04288 |
| O | 0.26924 | 2.13017 | -0.43209 | H | -0.27755 | 2.68678 | -0.99254 |
| O | -1.23316 | 0.39224 | 1.45172 | H | -1.33988 | -0.07728 | 2.28319 |
| O | 2.52955 | -1.55255 | -0.35459 | H | -1.52575 | 1.29733 | 1.58707 |
| H | -2.07073 | 0.29933 | -2.41818 | H | 3.21376 | -1.41152 | -1.01469 |
| H | -0.8472 | 0.91736 | -3.14811 | H | 2.71051 | -2.39034 | 0.07961 |
| H | 2.14951 | 1.29071 | -2.47117 | Pr | 0.29728 | -0.38213 | -0.43379 |

[Pr(H_2_O)_8_(ODSO_4_)]^2+^

| Atom | Coordinate (Å) | | | Atom | Coordinate (Å) | | |
| --- | --- | --- | --- | --- | --- | --- | --- |
|  | X | Y | Z |  | X | Y | Z |
| S | 5.21846 | 2.37713 | 0.03074 | H | -2.39745 | 1.8093 | 1.06716 |
| O | 6.16812 | 1.34661 | -0.415 | C | -3.55354 | 0.73125 | -0.38986 |
| O | 5.16233 | 2.47139 | 1.47584 | H | -3.39179 | -0.25788 | 0.05309 |
| O | 5.36227 | 3.62323 | -0.65383 | H | -3.56125 | 0.58322 | -1.47551 |
| O | 4.8501 | -2.61676 | 0.92283 | C | -4.90975 | 1.26324 | 0.05509 |
| O | 7.53612 | -2.15684 | 2.12164 | H | -5.06816 | 2.25758 | -0.37718 |
| H | 4.02027 | -2.7115 | 0.44648 | H | -4.90444 | 1.39922 | 1.1424 |
| H | 4.96277 | -3.39108 | 1.47959 | C | -6.07081 | 0.35644 | -0.33211 |
| O | 8.39752 | 0.40752 | 1.14764 | H | -5.90388 | -0.64247 | 0.08644 |
| O | 4.42111 | -0.90631 | -1.26018 | H | -6.08496 | 0.2336 | -1.42092 |
| O | 7.22455 | -0.45541 | -2.39423 | C | -7.42593 | 0.87413 | 0.13263 |
| O | 8.96016 | -1.82031 | -0.53254 | H | -7.58927 | 1.8777 | -0.27598 |
| O | 6.5289 | -3.05122 | -1.50807 | H | -7.41507 | 0.98541 | 1.22274 |
| H | 7.15714 | -2.00737 | 2.99198 | C | -8.58703 | -0.02637 | -0.26898 |
| H | 8.0952 | -2.93562 | 2.17746 | H | -8.41789 | -1.03307 | 0.12957 |
| H | 8.68892 | 0.20063 | 2.03984 | H | -8.60382 | -0.12803 | -1.35995 |
| H | 8.36431 | 1.36497 | 1.07092 | C | -9.94162 | 0.48075 | 0.20877 |
| H | 4.28258 | -1.24889 | -2.14628 | H | -10.10739 | 1.49126 | -0.18138 |
| H | 4.00207 | -0.03218 | -1.20877 | H | -9.92806 | 0.57256 | 1.30066 |
| H | 6.87992 | 0.37492 | -2.73629 | C | -11.10269 | -0.41389 | -0.20587 |
| H | 8.1569 | -0.48768 | -2.62918 | H | -10.93312 | -1.42648 | 0.17727 |
| H | 9.73852 | -1.43745 | -0.11882 | H | -11.12002 | -0.499 | -1.29826 |
| H | 9.18624 | -2.70777 | -0.82275 | C | -12.4571 | 0.08589 | 0.28005 |
| H | 6.08513 | -3.88396 | -1.33038 | H | -12.62389 | 1.10132 | -0.09668 |
| H | 6.66322 | -2.98981 | -2.45772 | H | -12.44242 | 0.16344 | 1.37302 |
| O | 3.84803 | 1.70709 | -0.4379 | C | -13.61819 | -0.8039 | -0.14489 |
| C | 2.60818 | 2.35861 | -0.07539 | H | -13.44904 | -1.82065 | 0.22723 |
| H | 2.54643 | 3.30586 | -0.61369 | H | -13.63519 | -0.87706 | -1.23814 |
| H | 2.62222 | 2.55287 | 0.99821 | C | -14.97256 | -0.30882 | 0.34602 |
| C | 1.47774 | 1.42933 | -0.44846 | H | -15.13972 | 0.70982 | -0.02184 |
| H | 1.51039 | 1.23568 | -1.52424 | H | -14.95731 | -0.24069 | 1.43963 |
| H | 1.62321 | 0.47241 | 0.06078 | C | -16.13377 | -1.19499 | -0.08573 |
| O | 5.56921 | -0.12598 | 2.15198 | H | -15.96601 | -2.21467 | 0.27937 |
| H | 4.76118 | -0.55953 | 2.44125 | H | -16.15103 | -1.26087 | -1.17959 |
| H | 5.38938 | 0.83756 | 2.13044 | C | -17.48892 | -0.70302 | 0.40777 |
| C | 0.12226 | 2.01269 | -0.06691 | H | -17.6544 | 0.31631 | 0.04453 |
| H | -0.0126 | 2.98015 | -0.56107 | H | -17.4713 | -0.63995 | 1.5005 |
| H | 0.10226 | 2.20823 | 1.01014 | C | -18.64234 | -1.59464 | -0.03287 |
| C | -1.03569 | 1.09302 | -0.43262 | H | -18.51404 | -2.61279 | 0.34272 |
| H | -0.88286 | 0.11612 | 0.03875 | H | -19.59976 | -1.22085 | 0.33435 |
| H | -1.0305 | 0.91772 | -1.51379 | H | -18.69967 | -1.64837 | -1.12281 |
| C | -2.393 | 1.6442 | -0.01598 | Pr | 6.61671 | -1.03222 | 0.02644 |
| H | -2.54237 | 2.62684 | -0.47686 |  |  |  |  |

[Sm(H_2_O)_9_]^3+^

| Atom | Coordinate (Å) | | | Atom | Coordinate (Å) | | |
| --- | --- | --- | --- | --- | --- | --- | --- |
|  | X | Y | Z |  | X | Y | Z |
| O | 0.07252 | -1.15198 | -2.18341 | H | -1.98151 | 1.72126 | 1.73327 |
| O | -1.64213 | -1.82599 | -0.05129 | H | -2.69518 | 1.16358 | -1.10902 |
| H | 0.54174 | -0.86136 | -2.96961 | H | -1.96473 | 0.65169 | -2.38466 |
| H | -0.51016 | -1.87242 | -2.43784 | H | 2.68477 | 0.33454 | -1.67775 |
| O | -1.93185 | 0.83995 | 1.35347 | H | 1.9846 | 1.71767 | -1.72931 |
| O | -1.88706 | 0.75752 | -1.43321 | H | 2.68581 | 1.19253 | 1.10972 |
| O | 1.92572 | 0.83156 | -1.36207 | H | 1.9568 | 0.67724 | 2.38368 |
| O | 1.87777 | 0.7854 | 1.4327 | H | -0.53336 | -0.87123 | 2.97948 |
| O | -0.06008 | -1.14718 | 2.19046 | H | 0.51963 | -1.87378 | 2.43396 |
| O | 1.65183 | -1.81765 | 0.06148 | H | 1.73668 | -2.43224 | -0.67291 |
| O | -0.00347 | 2.48094 | -0.01014 | H | 2.49395 | -1.8017 | 0.52445 |
| H | -2.48698 | -1.8133 | -0.50929 | H | 0.55146 | 3.04977 | 0.53017 |
| H | -1.72022 | -2.4412 | 0.68338 | H | -0.56818 | 3.04241 | -0.54804 |
| H | -2.68079 | 0.33803 | 1.685 | Sm | -0.00065 | 0.03319 | -0.00014 |

[Sm(H_2_O)_8_(ODSO_4_)]^2+^

| Atom | Coordinate (Å) | | | Atom | Coordinate (Å) | | |
| --- | --- | --- | --- | --- | --- | --- | --- |
|  | X | Y | Z |  | X | Y | Z |
| S | -5.09399 | 2.35296 | -0.01122 | H | 2.51671 | 1.73303 | -1.11064 |
| O | -6.04665 | 1.33515 | 0.45644 | C | 3.6804 | 0.69128 | 0.36675 |
| O | -5.05965 | 2.43433 | -1.45804 | H | 3.51991 | -0.30785 | -0.05363 |
| O | -5.21271 | 3.60419 | 0.66703 | H | 3.69139 | 0.56752 | 1.45541 |
| O | -5.28581 | -2.86585 | -0.93196 | C | 5.03405 | 1.2168 | -0.09343 |
| O | -7.76482 | -1.93584 | -2.061 | H | 5.19015 | 2.22141 | 0.31532 |
| H | -4.34427 | -2.92399 | -0.74699 | H | 5.02618 | 1.32748 | -1.18359 |
| H | -5.47594 | -3.42611 | -1.68802 | C | 6.19884 | 0.3231 | 0.31258 |
| O | -8.30533 | 0.58961 | -1.01809 | H | 6.03549 | -0.68545 | -0.08365 |
| O | -4.35326 | -0.99643 | 0.9116 | H | 6.21427 | 0.22443 | 1.40383 |
| O | -7.00679 | -0.33499 | 2.38531 | C | 7.55157 | 0.8359 | -0.1644 |
| O | -8.93761 | -1.69694 | 0.59401 | H | 7.71058 | 1.84918 | 0.22135 |
| O | -6.58629 | -2.93768 | 1.52178 | H | 7.53998 | 0.92246 | -1.25673 |
| H | -7.44929 | -1.85543 | -2.96467 | C | 8.71679 | -0.04999 | 0.25726 |
| H | -8.49816 | -2.55609 | -2.059 | H | 8.55302 | -1.06591 | -0.11954 |
| H | -8.73411 | 0.39927 | -1.85665 | H | 8.7332 | -0.12796 | 1.35019 |
| H | -8.23091 | 1.54357 | -0.93304 | C | 10.06919 | 0.45369 | -0.23023 |
| H | -4.13309 | -1.39803 | 1.75599 | H | 10.22901 | 1.47345 | 0.13773 |
| H | -3.91589 | -0.12882 | 0.8795 | H | 10.05646 | 0.52136 | -1.32389 |
| H | -6.67886 | 0.54998 | 2.57584 | C | 11.23464 | -0.42513 | 0.20542 |
| H | -7.87704 | -0.40135 | 2.78866 | H | 11.07172 | -1.44666 | -0.15624 |
| H | -9.74569 | -1.20408 | 0.43034 | H | 11.25039 | -0.48674 | 1.29942 |
| H | -9.15722 | -2.47571 | 1.11176 | C | 12.58702 | 0.07222 | -0.28858 |
| H | -6.12631 | -3.74519 | 1.27729 | H | 12.74668 | 1.09669 | 0.06616 |
| H | -6.57292 | -2.86937 | 2.48014 | H | 12.57431 | 0.12576 | -1.38302 |
| O | -3.72262 | 1.67101 | 0.43883 | C | 13.75267 | -0.80069 | 0.15829 |
| C | -2.48268 | 2.30909 | 0.04886 | H | 13.59102 | -1.82633 | -0.19212 |
| H | -2.41413 | 3.26824 | 0.5645 | H | 13.76731 | -0.85006 | 1.25292 |
| H | -2.50663 | 2.478 | -1.0288 | C | 15.1051 | -0.30752 | -0.33986 |
| C | -1.35147 | 1.38617 | 0.43431 | H | 15.26437 | 0.72013 | 0.00576 |
| H | -1.37772 | 1.21505 | 1.51387 | H | 15.09252 | -0.26374 | -1.43475 |
| H | -1.49979 | 0.41881 | -0.05416 | C | 16.27098 | -1.17599 | 0.11465 |
| O | -5.43772 | -0.17815 | -1.98763 | H | 16.1112 | -2.20465 | -0.22815 |
| H | -4.71885 | -0.65644 | -2.40668 | H | 16.28549 | -1.21745 | 1.20975 |
| H | -5.2364 | 0.78078 | -2.03311 | C | 17.62422 | -0.68582 | -0.38588 |
| C | 0.00166 | 1.96063 | 0.03111 | H | 17.78147 | 0.34261 | -0.04535 |
| H | 0.13924 | 2.93892 | 0.50266 | H | 17.60956 | -0.64762 | -1.47981 |
| H | 0.01528 | 2.13192 | -1.05013 | C | 18.78245 | -1.55908 | 0.07827 |
| C | 1.16198 | 1.04969 | 0.41087 | H | 18.66236 | -2.58641 | -0.27436 |
| H | 1.00776 | 0.0627 | -0.0385 | H | 19.73836 | -1.18707 | -0.29463 |
| H | 1.1621 | 0.89792 | 1.4956 | H | 18.83685 | -1.58757 | 1.16932 |
| C | 2.51671 | 1.5931 | -0.02396 | Sm | -6.66393 | -0.97974 | -0.02445 |
| H | 2.66613 | 2.58641 | 0.41344 |  |  |  |  |

[Gd(H_2_O)_9_]^3+^

| Atom | Coordinate (Å) | | | Atom | Coordinate (Å) | | |
| --- | --- | --- | --- | --- | --- | --- | --- |
|  | X | Y | Z |  | X | Y | Z |
| O | -1.65538 | -1.72881 | -0.44213 | H | 2.81015 | 0.26493 | -2.05407 |
| O | -0.91039 | 0.62784 | -2.23253 | H | 1.03036 | -2.04077 | -2.9755 |
| H | -1.81323 | -2.34713 | 0.27846 | H | -0.25186 | -2.58138 | -2.29409 |
| H | -2.51335 | -1.42007 | -0.74869 | H | -0.07353 | -1.79649 | 2.33717 |
| O | 1.96598 | 0.71333 | -1.93305 | H | 1.01188 | -2.7013 | 1.69312 |
| O | 0.32083 | -1.81093 | -2.36868 | H | 2.17922 | 0.09538 | 2.16221 |
| O | 0.37694 | -2.0032 | 1.5129 | H | 2.11117 | 1.44057 | 1.39235 |
| O | 2.06215 | 0.48401 | 1.28947 | H | 0.89043 | 2.67298 | -0.85953 |
| O | 0.45408 | 2.17674 | -0.15971 | H | -0.30612 | 2.69579 | 0.12042 |
| O | -1.02616 | 0.38743 | 1.61722 | H | -1.97975 | 0.4851 | 1.53719 |
| O | 2.54266 | -1.60728 | -0.47924 | H | -0.74593 | 0.98764 | 2.31448 |
| H | -1.1434 | 0.0205 | -2.94257 | H | 3.23799 | -1.45893 | 0.16924 |
| H | -1.62568 | 1.26534 | -2.14965 | H | 2.60086 | -2.52776 | -0.75164 |
| H | 1.64172 | 0.95332 | -2.80794 | Gd | 0.39697 | -0.32419 | -0.33756 |

[Gd(H_2_O)_8_(ODSO_4_)]^2+^

| Atom | Coordinate (Å) | | | Atom | Coordinate (Å) | | |
| --- | --- | --- | --- | --- | --- | --- | --- |
|  | X | Y | Z |  | X | Y | Z |
| S | 13.82999 | -4.25299 | 7.39348 | H | 15.62805 | -11.02772 | 10.6778 |
| O | 12.73393 | -3.31449 | 7.71163 | C | 15.54339 | -11.14573 | 12.82314 |
| O | 13.51927 | -5.07423 | 6.24426 | H | 14.48239 | -11.415 | 12.87283 |
| O | 15.10809 | -3.61781 | 7.37065 | H | 15.73447 | -10.49296 | 13.68222 |
| O | 8.86218 | -5.37985 | 8.2501 | C | 16.38761 | -12.40695 | 12.95273 |
| O | 8.55842 | -3.45616 | 6.09066 | H | 17.44841 | -12.13987 | 12.88867 |
| H | 9.02739 | -5.87146 | 9.06025 | H | 16.18563 | -13.06549 | 12.10052 |
| H | 7.91649 | -5.41328 | 8.08233 | C | 16.13996 | -13.17001 | 14.24761 |
| O | 10.98479 | -2.168 | 5.77937 | H | 15.07711 | -13.42736 | 14.31795 |
| O | 11.17725 | -4.8584 | 9.65454 | H | 16.35309 | -12.51399 | 15.09909 |
| O | 11.38383 | -1.80389 | 9.58049 | C | 16.97265 | -14.4395 | 14.37115 |
| O | 9.29931 | -1.06516 | 7.75917 | H | 18.03504 | -14.18416 | 14.28778 |
| O | 8.93971 | -3.00846 | 9.95017 | H | 16.74964 | -15.10101 | 13.52647 |
| H | 8.455 | -4.244 | 5.54975 | C | 16.73885 | -15.19235 | 15.67459 |
| H | 7.69884 | -3.02978 | 6.13933 | H | 15.67504 | -15.43997 | 15.76321 |
| H | 10.44933 | -2.38539 | 5.01011 | H | 16.97095 | -14.53294 | 16.51846 |
| H | 11.90257 | -2.13268 | 5.49602 | C | 17.56247 | -16.46821 | 15.79328 |
| H | 11.01882 | -4.60506 | 10.56873 | H | 18.62569 | -16.22226 | 15.69365 |
| H | 12.11821 | -5.08676 | 9.57043 | H | 17.32212 | -17.13223 | 14.95535 |
| H | 12.34324 | -1.88169 | 9.61306 | C | 17.34076 | -17.21243 | 17.10377 |
| H | 11.18944 | -0.87321 | 9.42464 | H | 16.27658 | -17.45247 | 17.20753 |
| H | 9.4048 | -0.563 | 6.9457 | H | 17.58815 | -16.55008 | 17.94099 |
| H | 8.44065 | -0.84097 | 8.12828 | C | 18.1575 | -18.49304 | 17.21868 |
| H | 8.01972 | -3.25395 | 10.07297 | H | 19.22111 | -18.25424 | 17.10655 |
| H | 9.15183 | -2.34065 | 10.60762 | H | 17.90388 | -19.15881 | 16.38606 |
| O | 13.76197 | -5.19739 | 8.67217 | C | 17.94523 | -19.23082 | 18.53437 |
| C | 14.64915 | -6.34423 | 8.72102 | H | 16.88102 | -19.46548 | 18.64943 |
| H | 15.67733 | -5.9797 | 8.71095 | H | 18.20384 | -18.5663 | 19.36644 |
| H | 14.47012 | -6.9541 | 7.83441 | C | 18.75719 | -20.51478 | 18.64645 |
| C | 14.34206 | -7.1059 | 9.98767 | H | 19.82098 | -20.28085 | 18.52593 |
| H | 14.48813 | -6.44646 | 10.84717 | H | 18.49454 | -21.18141 | 17.81732 |
| H | 13.29029 | -7.4061 | 9.97942 | C | 18.55128 | -21.24858 | 19.96525 |
| O | 10.81952 | -5.18518 | 6.34159 | H | 17.48719 | -21.48057 | 20.08804 |
| H | 10.43898 | -6.02611 | 6.61465 | H | 18.81689 | -20.58322 | 20.79464 |
| H | 11.76971 | -5.32699 | 6.14907 | C | 19.3604 | -22.53502 | 20.07644 |
| C | 15.22561 | -8.34068 | 10.12375 | H | 20.42275 | -22.30239 | 19.95127 |
| H | 16.27817 | -8.04029 | 10.10567 | H | 19.09258 | -23.19965 | 19.24887 |
| H | 15.07349 | -8.99399 | 9.2586 | C | 19.14962 | -23.25841 | 21.39999 |
| C | 14.94661 | -9.12213 | 11.40122 | H | 18.09913 | -23.52767 | 21.53462 |
| H | 13.88886 | -9.40541 | 11.4277 | H | 19.73933 | -24.17529 | 21.45305 |
| H | 15.11326 | -8.47099 | 12.26603 | H | 19.43963 | -22.62458 | 22.2416 |
| C | 15.80586 | -10.37218 | 11.53748 | Gd | 10.34017 | -3.34148 | 7.88467 |
| H | 16.86376 | -10.09038 | 11.49504 |  |  |  |  |

[Sc(H_2_O)_6_]^3+^

| Atom | Coordinate (Å) | | | Atom | Coordinate (Å) | | |
| --- | --- | --- | --- | --- | --- | --- | --- |
|  | X | Y | Z |  | X | Y | Z |
| O | 2.11418 | -0.04749 | -0.05398 | H | 0.70388 | -2.70907 | 0.04794 |
| H | 2.65225 | -0.04533 | -0.85417 | H | -0.85663 | -2.65103 | 0.04397 |
| H | 2.7075 | -0.04559 | 0.70651 | O | 0.04694 | 0.0547 | 2.11259 |
| O | -2.10765 | -0.0631 | -0.03777 | H | 0.04535 | -0.70566 | 2.70606 |
| H | -2.69493 | -0.06344 | 0.72734 | H | 0.04109 | 0.85494 | 2.65049 |
| H | -2.65315 | -0.06482 | -0.83304 | O | 0.06535 | 0.03963 | -2.10899 |
| O | -0.03845 | 2.10724 | 0.0642 | H | 0.06686 | 0.83656 | -2.65206 |
| H | -0.83447 | 2.65162 | 0.06336 | H | 0.06525 | -0.72367 | -2.69861 |
| H | 0.72587 | 2.69554 | 0.06427 | Sc | 0.00627 | -0.00667 | 0.00429 |
| O | -0.05552 | -2.11436 | 0.0476 |  |  |  |  |

[Sc(H_2_O)_5_(ODSO_4_)]^2+^

| Atom | Coordinate (Å) | | | Atom | Coordinate (Å) | | |
| --- | --- | --- | --- | --- | --- | --- | --- |
|  | X | Y | Z |  | X | Y | Z |
| O | -9.6013 | -2.441 | 1.5549 | H | 2.03687 | -0.94912 | -0.25566 |
| H | -9.42506 | -2.48113 | 2.50049 | C | 3.45152 | 0.66593 | -0.3716 |
| H | -10.15845 | -3.18687 | 1.31154 | H | 3.44808 | 0.70351 | -1.4667 |
| O | -8.67056 | 0.04969 | -1.68146 | H | 3.53105 | 1.70402 | -0.02976 |
| H | -8.97716 | -0.17725 | -2.56255 | C | 4.67239 | -0.11139 | 0.10332 |
| H | -8.03689 | 0.80802 | -1.70682 | H | 4.67839 | -0.13569 | 1.19889 |
| O | -7.34649 | -2.30577 | -0.4825 | H | 4.58937 | -1.15344 | -0.22532 |
| H | -7.02734 | -3.06345 | 0.01854 | C | 5.99127 | 0.46812 | -0.39178 |
| H | -6.66812 | -2.06772 | -1.12264 | H | 5.99984 | 0.46385 | -1.48753 |
| O | -10.07353 | -2.29297 | -1.13365 | H | 6.05969 | 1.51911 | -0.08913 |
| H | -9.69652 | -2.97533 | -1.69736 | C | 7.2124 | -0.28148 | 0.12528 |
| H | -10.99026 | -2.15805 | -1.39242 | H | 7.20593 | -0.26588 | 1.22101 |
| Sc | -8.90758 | -0.95376 | 0.16118 | H | 7.14092 | -1.33543 | -0.16607 |
| S | -6.64531 | 1.50907 | 0.34401 | C | 8.53257 | 0.28978 | -0.37589 |
| O | -6.58959 | 2.57876 | 1.28258 | H | 8.55132 | 0.25049 | -1.47082 |
| O | -7.58986 | 0.4336 | 0.78111 | H | 8.59223 | 1.35032 | -0.10651 |
| O | -6.9213 | 1.88523 | -1.02661 | C | 9.75319 | -0.43584 | 0.17545 |
| O | -5.30527 | 0.70705 | 0.37582 | H | 9.73643 | -0.38699 | 1.27008 |
| C | -4.10067 | 1.39481 | -0.05968 | H | 9.69067 | -1.49859 | -0.08434 |
| H | -4.18706 | 1.58275 | -1.13088 | C | 11.07446 | 0.12804 | -0.33113 |
| H | -4.0317 | 2.34573 | 0.47124 | H | 11.10052 | 0.06125 | -1.42459 |
| C | -2.92558 | 0.50296 | 0.25833 | H | 11.12821 | 1.19528 | -0.08825 |
| H | -2.89406 | 0.32645 | 1.33643 | C | 12.29439 | -0.57882 | 0.24555 |
| H | -3.06801 | -0.46575 | -0.22733 | H | 12.27021 | -0.5046 | 1.3386 |
| O | -10.80396 | 0.06286 | 0.25744 | H | 12.23814 | -1.64759 | 0.01014 |
| H | -11.42016 | 0.05063 | 0.99695 | C | 13.61644 | -0.02104 | -0.26581 |
| H | -11.02615 | 0.81142 | -0.30579 | H | 13.64647 | -0.10684 | -1.35786 |
| C | -1.61406 | 1.1279 | -0.20443 | H | 13.66709 | 1.0504 | -0.04145 |
| H | -1.50787 | 2.12403 | 0.23744 | C | 14.83586 | -0.71539 | 0.32665 |
| H | -1.64049 | 1.27058 | -1.28954 | H | 14.80808 | -0.62493 | 1.41853 |
| C | -0.40094 | 0.28463 | 0.16677 | H | 14.78408 | -1.78795 | 0.10732 |
| H | -0.51471 | -0.72018 | -0.2541 | C | 16.15916 | -0.16224 | -0.18805 |
| H | -0.36935 | 0.16132 | 1.25469 | H | 16.18821 | -0.25808 | -1.27814 |
| C | 0.91504 | 0.88539 | -0.30977 | H | 16.20737 | 0.9101 | 0.02676 |
| H | 0.89907 | 0.97206 | -1.40182 | C | 17.37098 | -0.85816 | 0.4175 |
| H | 1.00721 | 1.90603 | 0.07789 | H | 18.30426 | -0.4446 | 0.03122 |
| C | 2.13393 | 0.07713 | 0.11588 | H | 17.3809 | -0.74866 | 1.50473 |
| H | 2.15347 | 0.00647 | 1.2092 | H | 17.36001 | -1.92743 | 0.19233 |

**<Investigation of cation exchange reaction>**

The cation exchange reaction between solid-state surfactants and an aqueous solution of metal cations was investigated by immersing the surfactant crystals, including only Gd as the counterion of the surfactants, in the solution of cerium nitrate. EDS spectrum and EDS mappings clearly showed that Ce cations were incorporated in solid-state surfactants after immersing, indicating the cation exchange reaction after the precipitation process (Figure S10).

**Figure S10.** SEM images, EDS mappings and EDS spectra of solid-state surfactants before/after the immersion in aqueous solution of cerium nitrate.

**<Thermodynamic stability of the solid-state surfactants>**

Solid-state surfactants have a lamellar structure in which metal cations are arranged two-dimensionally in the interlayer space between surfactant layers formed by the two-dimensional assembly of surfactant molecules through van der Waals interactions. In these surfactant layers, the alkyl chains are known to pack in either hexagonal or orthorhombic arrangements, with a characteristic interchain spacing of approximately 0.4 nm. In particular, in the *L*_c_ phase relevant to the present system, the alkyl chains have little rotational freedom, and their conformations are highly restricted.^[9,10]^ Under such conformational constraints, the distance between the hydrophilic groups that interact with the cations is also restricted, giving rise to a certain degree of electrostatic repulsion between the negatively charged headgroups.

Based on this structural picture, we considered the incorporation of trivalent cations with different ionic radii into the interlayer space of the solid-state surfactant. When a trivalent cation is introduced, charge neutrality requires coordination of three surfactant anions per cation, and at least two of the corresponding alkyl chains are expected to remain in a packed state within the lamellar assembly. Under these conditions, introduction of a smaller cation is expected to draw the coordinating headgroups more strongly toward the cation center, thereby decreasing the distance between the charged groups. This structural contraction increases the electrostatic repulsion between the headgroups and is therefore expected to reduce the thermodynamic stability of the solid-state surfactant as a whole (Figure S11). Consequently, when cations of the same valence are compared, those with smaller ionic radii are expected to form less stable solid-state surfactants and to be more readily excluded through cation exchange.

**Figure S11.** Illustration of the effect of cation size on electrostatic repulsion between hydrophilic headgroups.

**Figure S12.** Metal compositions in solid-state surfactants synthesized with various preparation conditions. (a) Relationship between preparation amount and induced amount of Fe in the solid-state surfactants synthesized using Ga and Fe as cations investigated by EDXRF. (b) Relationship between preparation amount and induced amount of Ga in the solid-state surfactants synthesized using La and Ga as cations investigated by ICP-AES.

**<Characterization of intermediate crystals >**

The structure and composition of the intermediates synthesized with all preparation ratios were analyzed by XRD, EDXRF, and SEM-EDS. In addition, we carried out detailed analyses for the intermediates synthesized with the preparation ratio of Ce:Gd = 0.70:0.30 by TEM, FT-IR, TG, and CHNS. In the XRD patterns of the intermediates, there were peaks in the 2*θ* range of 1–10°, indicating the presence of lamellar structures (Figures S13–S15). For example, the XRD pattern of the intermediate prepared with the starting ratio of Ce:Gd = 0.70:0.30 showed the sharp peaks at 2*θ* = 1.5° and 3.1° (The *d*-values were 5.8 nm and 2.8 nm.), and broad peaks at 2*θ* = 2.1°, 4.2°, and 6.3° (The *d*-values were 4.2 nm, 2.1 nm, and 1.4 nm, respectively.), differing from the precursor's *d*-spacing of 5.2 nm. The sharp peaks were attributed to ammonium octadecylsulfate. Furthermore, there were broad diffraction peaks at 2*θ* = 28.5°, 33.1°, 47.5°, and 56.1°, confirming the formation of ceria with a fluorite structure. Notably, no diffraction peaks corresponding to Gd oxide/hydroxide were detected, suggesting selective ceria formation through humid ammonia vapor treatment (Figure S13). Furthermore, the TEM image of the intermediate synthesized with the preparation ratio of Ce:Gd = 0.70:0.30 showed the periodic contrast composed of nanoparticles. In addition, lattice fringes with spacings of 0.32 nm and 0.28 nm were observed, which closely correspond to the {111} and {200}, respectively, of fluorite-structured ceria (Figure S16). These data suggested the formation of ammonium octadecylsulfate and RE-doped ceria.

The metal compositions were investigated by EDXRF and SEM-EDS for all samples. The EDXRF spectra showed the peaks assigned to Ce and RE elements (Figures S17 and S18). The calculated metal ratios of the intermediates containing Ce and Gd were Ce:Gd = 0.93:0.07 (metal ratio in the precursors; Ce:Gd = 0.93:0.07), 0.85:0.15 (0.85:0.15), 0.77:0.23 (0.77:0.23), 0.68:0.32 (0.69:0.31), and 0.60:0.40 (0.59:0.41), as shown in Figure 3d. For other dopants, metal molar ratios were Ce:La = 0.67:0.33 (metal ratio in the intermediates; Ce:La = 0.67:0.33), Ce:Pr = 0.72:0.28 (0.72:0.28), Ce:Sm = 0.78:0.22 (0.78:0.22), Ce:Yb = 0.80:0.20 (0.83:0.17), Ce:Y = 0.85:0.15 (0.89:0.11), and Ce:Sc = 0.91:0.09 (0.93:0.07), as shown in Figure 3e. This indicated the metal compositions in the intermediates almost corresponded to those of the precursors. EDS mapping showed the homogeneous distribution of Ce, RE, and sulfur throughout the crystal. The uniform distribution of RE indicates no detectable segregation of RE oxide or hydroxide at the EDS resolution (Figures S19 and S20).

Then, to confirm the retention of the original surfactant structure, we investigated the molecular structures of the intermediate. FT-IR showed characteristic peaks derived from sulfate groups (1060 cm⁻^1^) and alkyl groups (2848 cm⁻^1^, 2917 cm⁻^1^, and 2955 cm⁻^1^), suggesting that the molecular structure of the surfactants was maintained after humid ammonia vapor treatment. Additionally, a peak associated with N–H stretching vibrations appeared at 3078 cm⁻¹, corresponding to the formation of ammonium octadecylsulfate (Figure S21a).

The composition of the resulting crystals was precisely examined by combining EDXRF, CHNS and TG analyses (Figure S21b). For instance, the composition of the intermediate synthesized by using the precursor with the metal composition of Ce:Gd = 0.77:0.23 was calculated to be C_18_H_37_SO_4_·0.11C_18_H_37_OH·0.38Ce_0.77_Gd_0.23_OₓH_y_·NH_4_·0.31H_2_O (Table S1).

**Figure S13.** XRD patterns of the intermediate synthesized with the preparation ratio of Ce:Gd = 0.70:0.30. The orange and blue markers represent peak positions corresponding to ammonium octadecylsulfate and fluorite-structured ceria, respectively.

**Figure S14.** XRD patterns of intermediates synthesized by using Ce and Gd with the various preparation ratios of Ce:Gd = 0.90:0.10, 0.80:0.20, 0.60:0.40, and 0.50:0.50.

**Figure S15.** XRD patterns of intermediates synthesized by using Ce and RE (RE = La, Pr, Sm, Yb, Y, or Sc) with the preparation ratio of Ce:RE = 0.70:0.30.

**Figure S16.** TEM images and FFT patterns of the intermediate synthesized with the preparation ratio of Ce:Gd = 0.70:0.30.

**Figure S17.** XRF spectra of intermediates synthesized by using Ce and Gd with the various preparation ratios of Ce:Gd = 0.90:0.10, 0.80:0.20, 0.60:0.40, and 0.50:0.50.

**Figure S18.** EDXRF spectra of intermediates synthesized by using Ce and RE (RE=La, Pr, Sm, Yb, Y, or Sc) with the preparation ratio of Ce:RE = 0.70:0.30.

**Figure S19.** SEM images, EDS mappings, and EDS spectra of intermediates synthesized by using Ce and Gd with the various preparation ratios of Ce:Gd = 0.90:0.10, 0.80:0.20, 0.60:0.40, and 0.50:0.50.

**Figure S20.** SEM images, EDS mappings, and EDS spectra of intermediates synthesized by using Ce and RE (RE=La, Pr, Sm, Yb, Y, or Sc) with the preparation ratio of Ce:RE = 0.70:0.30.

**Figure S21.** (a) FT-IR spectrum, and (b) TG curve of intermediates synthesized with the preparation ratio of Ce:Gd = 0.70:0.30.

**Figure S22.** AFM images with height profiles (solid line) and the corresponding evaluated places (dashed line) of RE-doped ceria nanosheets (RE = La, Pr, Sm, Yb, Y, and Sc).

**Figure S23.** TEM images and (inset) SAED patterns, EDS mappings, and EDS spectra of Gd-doped ceria nanosheets synthesized with various preparation ratios.

The peaks at 1.7 keV (Si Kα), 6.4 keV (Fe Kα), and 6.9 keV (Co Kα) are derived from the TEM machine and EDS detector.

The peak at 8.0 keV (Cu Kα) is derived from the TEM grid.

**Figure S24.** TEM images and (inset) SAED patterns, EDS mappings, and EDS spectra of RE-doped ceria nanosheets (RE=La, Pr, Sm, Yb, Y, and Sc).

**Figure S25.** Comparison between the SAED pattern of the nanosheet synthesized with the preparation ratio of Ce:Gd = 0.7:0.3 and the Debye ring simulated by ReciPro.^[11]^

**<Long-term stability of Gd-doped ceria nanosheet>**

To evaluate the long-term stability of the nanosheets, a colloidal dispersion of nanosheets prepared with a preparation ratio of Ce:Gd = 0.70:0.30 was analyzed after being stored under ambient conditions for more than four months. TEM observations confirmed the presence of nanosheets, and SAED measurements showed that they retained a polycrystalline ceria structure (Figure S26). Furthermore, SEM-EDS analysis detected both Ce and Gd in the nanosheets, with a composition of Ce:Gd = 0.90:0.10 (SD = 0.011 for Gd), indicating that the composition was largely preserved during storage. These results demonstrate that the nanosheets obtained by this method possess robust chemical and structural stability over this period.

**Figure S26.** The nanosheets stored under ambient conditions for more than four months. (a) TEM image and (inset) SAED pattern, (b) SEM image, EDS mappings, and EDS spectrum of nanosheets.

**< Composition control of GaFeO*_x_* nanosheets>**

GaFeO*_x_* nanosheets were synthesized using the same procedure as that employed for the rare-earth-doped ceria nanosheets. Nanosheets were successfully obtained when solid-state surfactants with Ga:Fe ratios of 0.74:0.26 and 0.56:0.44 were used as precursors. Their morphology and composition were analyzed by SEM-EDS (n = 3). SEM images revealed sheet-like structures similar to those observed for the rare-earth-doped ceria nanosheets. EDS analysis confirmed that these sheet-like products contained both Ga and Fe (Figure S27a). The compositions of the nanosheets were determined to be Ga:Fe = 0.76:0.24 (metal ratio in the solid-state surfactant: Ga:Fe = 0.74:0.26) and 0.60:0.40 (0.56:0.44), with standard deviations for the Fe fraction of 0.007 and 0.01, respectively (Figure S27b). These results indicate that more than 90% of the Fe present in the precursor solid-state surfactants was incorporated into the nanosheets, demonstrating that most of the Fe was retained during nanosheet formation. This higher incorporation efficiency than that observed for the rare-earth elements is likely due to the greater tendency of Ga^3+^ and Fe^3+^ to undergo hydrolysis, which would promote nearly complete precipitation as oxide/oxyhydroxide species during the ammonia vapor treatment.

**Figure S27.** Characterization of GaFeO*_x_* nanosheets. (a) SEM images, EDS mappings, and EDS spectra of GaFeO*_x_* nanosheet synthesized using solid-state surfactants with different Ga:Fe composition. (b) The amounts of Fe in the nanosheets compared to those of precursor solid-state surfactants investigated by SEM-EDS.

**Figure S28.** Comparison of XPS spectra of ceria nanosheets: CeO_2−_*_x_* (0%), Ce_0.87_Gd_0.13_O_2-_*_x_* (13%), and Ce_0.78_Gd_0.22_O_2-_*_x_* (22%) nanosheets. Changes in the intensity of v’ peaks associated with the Gd content were observed in the region highlighted by the red dashed lines.

**<Conductivity measurement of Gd-doped ceria nanosheet>**

For characterization of the Ce_0.90_Gd_0.10_O_2−_*_x_* nanosheets, electrode devices were fabricated on individual nanosheets according to a previously reported procedure.^[12]^ First, Ce_0.90_Gd_0.10_O_2−_*_x_* nanosheets were transferred onto a sapphire substrate. The substrate was then calcined in air at 500 °C for 12 h, followed by ultrasonication in acetone, ethanol, and water for 30 min each to remove residual surfactant species and adsorbed water from the substrate and nanosheets. Next, a photoresist layer was formed by spin-coating AZ1500 photoresist polymer onto the substrate at 5000 rpm for 60 s, followed by heating at 100 °C for 3 min. After preparation of the photoresist layer, the positions of individual nanosheets were identified by optical microscopy. Comb-like electrode patterns were then projected under safe red light to align the designated pattern with the nanosheets, and the selected area was exposed to blue light from an LED projector for 0.1 s. After exposure, the substrate was immersed in NMD-3 (2.38%) for 10 s and rinsed with ultrapure water. The patterned substrate was subsequently coated with a 25 nm Pt layer using a quick coater (Sanyu Electron, SC-701 MkII). The substrate was then treated with UV-Ozone for 15 min to improve the adhesion between the substrate and the Pt layer.^[13]^ Finally, the substrate was ultrasonicated in acetone for 1 min to lift off the excess metal, thereby completing fabrication of the nanosheet device shown in Figures S29a and S29b. The electrical conductivity of the fabricated nanosheet devices was evaluated by applying an AC voltage using an impedance analyzer. The measurements were conducted over a frequency range from 1 Hz to 1 MHz with an applied voltage of 50 mV. As a result of the impedance measurements, clear Nyquist plots were obtained, indicating that the Ce_0.90_Gd_0.10_O_2−_*_x_* nanosheet exhibits conductive behavior (Figures S30a and S30b). The ionic conductivity was significantly lower than that of pristine ceria.^[14]^ This difference suggests that the ionic conductivity of pristine ceria nanosheets may be associated with Ce^3+^-related conduction pathways, which are perturbed by Gd incorporation. Together with XPS data, our preliminary conductivity measurements indicate that, while Gd doping effectively modifies the redox state and defect population, it simultaneously suppresses vacancy mobility, leading to reduced ionic conductivity. In Ce_0.90_Gd_0.10_O_2−_*_x_* nanosheets, the increase in vacancy concentration is outweighed by a reduction in vacancy mobility arising from dopant–vacancy association, electronic conduction contribution, and pronounced surface/interface effects inherent to the 2D geometry. Although a comprehensive understanding of Gd doping effects requires further detailed characterization, the present results already demonstrate the functional relevance of controlled doping.

**Figure S29.** The nanosheet device fabricated by an LED-lithography-assisted method. (a) Confocal laser microscope image, and (b) AFM image of the electrode with the Ce_0.90_Gd_0.10_O_2−_*_x_* nanosheet.

**Figure S30.** Conductivity measurement of the Ce_0.90_Gd_0.10_O_2−_*_x_* nanosheet. (a) Nyquist plots obtained by electrochemical impedance measurements. (b) Conductivity of the Ce_0.90_Gd_0.10_O_2−_*_x_* and CeO_2−_*_x_* nanosheets. The conductivity data of CeO_2−_*_x_* nanosheet were taken from our previous report.^[14]^

**References**

[1]	M. J. Frisch, G. W. Trucks, H. B. Schlegel, et al., Gaussian 16, Revision C.01, Gaussian, Inc., Wallingford CT, 2016.

[2]	J.-D. Chai, and M. Head-Gordon, “Long-range corrected hybrid density functionals with damped atom–atom dispersion corrections,” *Physical Chemistry Chemical Physics* 10 (2008): 6615–6620, https://doi.org/10.1039/B810189B.

[3]	F. Weigend, and R. Ahlrichs, “Balanced basis sets of split valence, triple zeta valence and quadruple zeta valence quality for H to Rn: Design and assessment of accuracy” *Physical Chemistry Chemical Physics* 7 (2005): 3297–3305, https://doi.org/10.1039/B508541A.

[4]	F. Martelli, S. Abadie, J.-P. Simonin, R. Vuilleumier, and R. Spezia, “Lanthanoids(III) and actinoids(III) in water: Diffusion coefficients and hydration enthalpies from polarizable molecular dynamics simulations” *Pure and Applied Chemistry* 85 (2012): 237–246, https://doi.org/10.1351/PAC-CON-12-02-08.

[5]	J. Zhang, N. Heinz, and M. Dolg, “Understanding Lanthanoid(III) Hydration Structure and Kinetics by Insights from Energies and Wave functions” *Inorganic Chemistry* 53 (2014): 7700–7708, https://doi.org/10.1021/ic500991x.

[6]	E. Cancès, B. Mennucci, and J. Tomasi, “A new integral equation formalism for the polarizable continuum model: Theoretical background and applications to isotropic and anisotropic dielectrics” *The Journal of Chemical Physics* 107 (1997): 3032–3041, https://doi.org/10.1063/1.474659.

[7]	M. Cossi, N. Rega, G. Scalmani, and V. Barone, “Energies, structures, and electronic properties of molecules in solution with the C-PCM solvation model” *Journal of Computational Chemistry* 24 (2003): 669–681, https://doi.org/10.1002/jcc.10189.

[8]	V. M. Coiro, M. Manigrasso, F. Mazza, and G. Pochetti, “Structure of a triclinic phase of sodium dodecyl sulfate monohydrate. A comparison with other sodium dodecyl sulfate crystal phases” *Acta Crystallographica Section C Crystal Structure Communications* 43 (1987): 850–854, https://doi.org/10.1107/S010827018709382X.

[9]	K. Takeda, Y. Andoh, W. Shinoda, and S. Okazaki, “Structure of Hydrated Crystal (L_c_), Tilted Gel (L*_β′_*), and Liquid Crystal (L*_α_*) Phases of Linear Alkylbenzene Sulfonate (LAS) Studied by X-ray Diffraction and Molecular Dynamics Simulation” *Langmuir* 35 (2019): 9011–9019, https://doi.org/10.1021/acs.langmuir.9b01199.

[10]	L. A. Smith, R. B. Hammond, K. J. Roberts, D. Machin, and G. McLeod, “Determination of the crystal structure of anhydrous sodium dodecyl sulphate using a combination of synchrotron radiation powder diffraction and molecular modelling techniques” *Journal of Molecular Structure* 554 (2000): 173–182, https://doi.org/10.1016/S0022-2860(00)00666-9.

[11]	Y. Seto, and M. Ohtsuka, “ReciPro: free and open-source multipurpose crystallographic software integrating a crystal model database and viewer, diffraction and microscopy simulators, and diffraction data analysis tools” *Journal of Applied Crystallography* 55 (2022): 397–410, https://doi.org/10.1107/S1600576722000139.

[12]	Y. Shi, T. Taniguchi, K.-N. Byun, et al., “Damage-free LED lithography for atomically thin 2D material devices” *Scientific Reports* 13 (2023): 2583, https://doi.org/10.1038/s41598-023-29281-w.

[13]	H. Le-The, R. M. Tiggelaar, E. Berenschot, A. van den Berg, N. Tas, and J. C. T. Eijkel, “Postdeposition UV-Ozone Treatment: An Enabling Technique to Enhance the Direct Adhesion of Gold Thin Films to Oxidized Silicon” *ACS Nano* 13 (2019): 6782–6789, https://doi.org/10.1021/acsnano.9b01403.

[14]	E. Yamamoto, D. Kurimoto, K. Ito, K. Hayashi, M. Kobayashi, and M. Osada, “Solid-state surfactant templating for controlled synthesis of amorphous 2D oxide/oxyhydroxide nanosheets” *Nature Communications* 15 (2024): 6612, https://doi.org/10.1038/s41467-024-51040-2.
